# Evaluating the Efficacy of Target Capture Sequencing for Genotyping in Cattle

**DOI:** 10.3390/genes15091218

**Published:** 2024-09-18

**Authors:** Yan Ren, Mehar S. Khatkar, Callum MacPhillamy, Haofei Wang, Rudi A. McEwin, Tong Chen, Wayne S. Pitchford, Wai Yee Low

**Affiliations:** 1 Davies Livestock Research Centre, School of Animal and Veterinary Sciences, University of Adelaide, Adelaide, SA 5371, Australia; kelly.ren@adelaide.edu.au (Y.R.); callum.macphillamy@adelaide.edu.au (C.M.); tong.chen@adelaide.edu.au (T.C.);; 2 MGI Australia Pty Ltd., Brisbane, QLD 4000, Australia

**Keywords:** targeted captured sequencing, liquid probe, cattle, animal breeding

## Abstract

(1) Background: Target capture sequencing (TCS) is potentially a cost-effective way to detect single-nucleotide polymorphisms (SNPs) and an alternative to SNP array-based genotyping. (2) Methods: We evaluated the effectiveness and reliability of TCS in cattle breeding scenarios using 48 female and 8 male samples. DNA was extracted from blood samples, targeted for 71,746 SNPs with TWIST probes, and sequenced on an MGI platform. GATK and BCFtools were evaluated for the best genotyping calling tool. The genotypes were compared to existing genotypes from the Versa50K SNP array of the same animals by measuring accuracy as concordance (%) and R^2^. (3) Results: In this study, 71,553 SNPs and 166 indels were identified. The genotype comparison of 37,130 common SNPs between TCS and SNP arrays yielded high agreement, with a mean concordance of 98%, R^2^ of 0.98 and Cohen’s kappa of 0.97. The concordances of sex prediction, parent verification and validation of five genotype markers of interest important for Wagyu breeding were 100% between TCS and SNP array. The elements of the genomic relationship matrix (GRM) constructed from the SNP array and TCS data demonstrated a correlation coefficient approaching unity (r = 0.9998). (4) Conclusions: Compared to the SNP array, TCS is a comparable, cost-effective and flexible platform for genotyping SNPs, including non-model organisms and underrepresented commercial animal populations.

## 1. Introduction

Genotyping with whole-genome sequencing (WGS) at a high coverage is ideal, but it is still costly to genotype many samples required in livestock breeding. Instead of sequencing the entire genome, some researchers have been selectively enriching specific genomic regions for sequencing, which reduces costs and allows for the accurate detection of genotypes [1,2]. Many methods have been applied in the past, including reduced-representation libraries (RRLs), complexity reduction of polymorphic sequences (CRoPSs), restriction-site-associated DNA sequencing (RAD-Seq), genotyping-by-sequencing (GBS) and genotyping-in-thousands by sequencing (GT-seq) [3,4,5,6]. Some of the methods, such as RRLs and CRoPS, utilising barcode-identifying sequences, were developed during the Sanger sequencing era and later adapted for next-generation sequencing. Both RAD-Seq and GBS rely on restriction enzymes for target sequencing, whereas GT-seq uses custom amplicon sequencing to enrich the regions of interest. 

Target capture technologies (TCS) are utilising hybridisation-based capture around known single-nucleotide polymorphic loci (SNPs), followed by short-read sequencing at a high coverage [7,8,9]. This method involves the use of capture probes to isolate the DNA fragments surrounding SNPs before sequencing, allowing for the cost-effective and accurate genotyping of various species, including non-model organisms [2,10]. The TWIST silicon platform technology, developed by Twist Bioscience, has enabled gene or sequence-specific enrichment through a hybridisation step with probes complementary to the regions of interest [11]. Comparing hybrid capture to amplicon-based methods, studies have demonstrated that hybrid capture provides a more uniform and deeper coverage, as well as higher sensitivity for variant calling compared to amplicon sequencing [12,13]. Additionally, hybrid capture has been found to result in fewer dropouts in sequencing data when compared to amplicon-based methods [14]. 

TCS has been used in genome sequencing for clinical diagnosis [15,16,17,18,19] and plant genotyping, including soybean [20], wheat [10,21], tomato [22] and maize [23], but its utilisation in animal studies is in early stages [7,24,25]. To isolate or enrich sequences of interest for TCS, the sequences need to be determined beforehand to generate a probe list, which is equivalent to an SNP panel in the context of SNP array genotyping. For instance, the 1000 Bull Genomes Project [26] and the 1000 Buffalo Genomes Project utilised short-read WGS to provide a comprehensive list of SNPs for cattle, and probe lists can be made from the discovered SNPs. If a large-scale initiative to discover SNPs for the species of interest is not available, it is possible to discover SNPs with low-pass WGS followed by validation of the SNPs before using them to generate a probe list. Usually, the number of SNPs discovered from WGS will be more than necessary for genotyping for applications in animal breeding scenarios and strategies to prioritise SNPs, typically based on the inter-probe distance and SNP allele frequency, will be required. 

In the context of cattle breeding, the use of genotype information has been shown to be useful for genomic prediction using BayesR and GBLUP models [27]. Genotype information is also useful to select for certain genotypes that are responsible for particular traits or inherited conditions, e.g., Fading Calf Syndrome, Chediak Higashi Syndrome, Claudin 16 Deficiency, Band 3 Spherocytosis, and Factor XI deficiency. Moreover, associations between specific SNP genotypes and traits related to meat quality, such as marbling, have been reported, highlighting the potential of using genotype in enhancing breeding strategies for improved beef quality [28]. By genotyping embryos, breeders can potentially perform preimplantation genomic selection to select for desirable traits [29]. Genotype information is also useful for parent verification [30,31,32], breed identification [33], the detection of selection signatures [34,35], genome-wide association studies (GWAS) [36] and expression quantitative trait loci (eQTL) [37]. 

The objective of this study was to comprehensively assess the concordance between our newly developed TWIST-based TCS 70K SNP panel and SNP array of cattle samples. We evaluated the sequencing data produced by TCS and conducted a comparison of the accuracy of genotypic calls produced by GATK and BCFtools using quality-controlled genotypes from the Versa50K Illumina SNP chip as the reference. Finally, we compared the genomic relationship matrix (GRM) derived from TCS and SNP chip genotypes, demonstrating potential for genomic selection.

## 2. Materials and Methods

### 2.1. Sampling and DNA Extraction

Fifty-six animals, including forty-eight females and eight males of a different batch, were randomly selected from a population of pure-bred Wagyu cattle for TCS. These samples were already genotyped on the Versa50K SNP array. DNA was extracted from the blood collected in the BD Vacutainer EDTA tube. The genomic deoxyribonucleic acid (gDNA) input has OD260/OD280 between 1.8 and 2.0 and ~30–120 ng in an average of 30 μL. The PCR-free workflow was used for library preparation, and the data yield after ligation is shown in Table S1. The male samples were sequenced separately and used to confirm the Y chromosome probes.

### 2.2. Target SNP Probe Design and Sequencing

By combining SNPs from an Illumina-based Versa50K and GGPLD30K chips, a panel of 80,989 SNPs was selected as targets for probe capture-based sequencing. The rationale behind using SNPs from Versa50K and GGPLD30K was due to the availability of Wagyu samples already genotyped on these SNP arrays, which allowed for comparison with other genotyping technologies. After removing the probes that were in highly repetitive regions or contained ambiguous bases using TWIST proprietary QC pipeline, a total of 71,655 probes remained. The average distance between those probes was ~74 kb, targeting a total of 71,746 SNPs. Each probe has a length of 120 base pairs (bp) and was designed to target regions of ~250 bp in size. A small number of probes targeted multiple SNPs. Library preparation was performed following Twist enzymatic fragmentation method, followed by standard hybridization and wash. For female samples, forty-eight fragmented samples were pooled, i.e., 48-plex, for probe hybridisation to capture and amplify target regions. For male samples, 8-plex was carried out. Genomic regions targeted by the probes were sequenced using MGI DNBSEQ G400 (MGI, Brisbane, Australia) to generate at least 30 millions of 2 × 150 bp reads per sample.

### 2.3. Bioinformatic Pipeline for TCS Data

The process of analysing TCS data are shown in Figure S1. The raw reads were cleaned by trimming 10 bp from the 5′ ends, filtered for Q-score > 30 and length > 75 bp with trim_galore v0.4.2 [38]. The alignment was carried out by bwa v0.7.17 [39] using the reference *Bos taurus* UMD3.1 (bostau6) plus a Wagyu Y chromosome from ARS-UCD2.0 [40]. When necessary, the aligned reads were visualised using the Integrative Genomics Viewer [41] to assess the sequencing depth of the intended regions. The coverage at a particular probe was calculated using function samtools bedcov. Subsequently, both BCFtools v1.9 [42] and Genome Analysis Toolkit (GATK v4.1.6) [43] were used for genotype calling. For BCFtools, the function samtools mpileup was used to generate text pileup output on binary alignment map (BAM) files. The genotypes of targeted regions were called by bcftools call. The function bcftools filter annotated the sites of expression depth lower than 100 and a QUAL value higher than 20. The GATK best practices pipeline [44] was implemented and we tested the SNP with and without base-call quality score modifications.

### 2.4. Processing SNP Array Data

The Versa50K SNP array final reports of genotypes for the same 56 animals were retrieved from our Wagyu cattle database. The genotypes of these 56 animals had an average call rate of ~0.98 (Table S2). The duplicated SNPs, for the same positions, were resolved by selecting the higher call rate SNP and where there was a tie, one of the SNPs was randomly selected. The genotypes were converted to a count of B alleles using 0, 1 and 2 to represent homozygous for the reference allele (AA), heterozygous (AB) and homozygous for the alternate allele (BB), respectively. The converted genotype matrix was later used to compare with the genotypes from TCS data.

To perform the quality control (QC) of SNPs for comparison with TCS, 332 trios genotyped (Table S3) with the Versa50K SNP array from the same population were selected for testing Mendelian consistency. SNPs exhibiting high Mendelian inconsistency (MI) on the Versa50K platform were excluded from the comparisons. Mendelian consistency refers to the adherence of genotypes to Mendel’s laws of inheritance within a family pedigree. Several studies have highlighted the importance of ensuring Mendelian consistency in genotype, calling to identify and correct genotyping errors [45,46]. 

### 2.5. Comparison of Genotypes between TCS and the SNP Array 

Only the common SNPs, which were 37,130 SNPs, between TCS and the Versa50K SNP array were used to assess the accuracy of genotyping. The concordance and R^2^ between genotypes from TCS and the SNP array were conducted at the sample and SNP levels. Specifically, the VCF file containing the genotypes for 56 samples was obtained and subsequently filtered to only use common SNPs that were present in the SNP array data. The data were in a matrix format, with the samples as columns and SNPs as rows. The genotypes in the matrix were formatted as the B allele count to be compatible with the SNP array matrix. Concordance analysis was simply the count of matching genotypes between TCS and the SNP array, and where there was a mismatch, the count of one-allele or two-allele differences was also computed. The coefficient of determination (R^2^) across all samples and across all SNPs was computed as follows.

The R^2^ for each sample, k, was calculated using the following formula:

$$R^{2}\left(sample\right)={\left(\frac{\sum_{i=1}^{n}\left({tg}_{ik}-\bar{tg_{k}}\right)\left({cg}_{ik}-\bar{{cg}_{k}}\right)}{\sqrt{\sum_{i=1}^{n}{\left({tg}_{ik}-\bar{{tg}_{k}}\right)}^{2}\sum_{i=1}^{n}{\left({cg}_{ik}-\bar{{cg}_{k}}\right)}^{2}}}\right)}^{2}$$

where n is the number of SNPs. ${tg}_{ik}$ and ${cg}_{ik}$ denote the genotypes for the i-th SNP of the k-th sample from TCS and the SNP chip, respectively. $\bar{tg_{k}}=\frac{1}{n}\sum_{i=1}^{n}{tg}_{ik}$ is the mean of genotypes for the k-th sample from TCS and analogously $\bar{{cg}_{k}}$ for the SNP chip.

The R^2^ for each SNP, i, was calculated as per the following formula:

$$R^{2}(SNP)={\left(\frac{\sum_{k=1}^{m}\left({tg}_{ik}-\bar{{tg}_{i}}\right)\left({cg}_{ik}-\bar{{cg}_{i}}\right)}{\sqrt{\sum_{k=1}^{m}{\left({tg}_{ik}-\bar{{tg}_{i}}\right)}^{2}\sum_{k=1}^{m}{\left({cg}_{ik}-\bar{{cg}_{i}}\right)}^{2}}}\right)}^{2}$$

where m is the total number of samples. ${tg}_{ik}$ and ${cg}_{ik}$ denote the genotypes for the i-th SNP of the k-th sample from TCS and the SNP chip, respectively. $\bar{{tg}_{i}}=\frac{1}{m}\sum_{k=1}^{m}{tg}_{ik}$ for the mean of the genotypes for the i-th SNP from TCS and analogously $\bar{{cg}_{i}}$ for the SNP chip. The lists of SNPs with less than 90% of concordance and R^2^ < 0.8 were identified. In addition, Cohen’s kappa coefficient, a measure of inter-rater agreement, was calculated for each sample across all common SNPs between TCS and SNP chip platforms using the *irr* R package [47]. Specifically, we employed the kappa2 function with squared weighting (weight = “squared”) to account for the ordinal nature of genotype data.

### 2.6. Identification of Problematic Regions and SNPs

The distribution of successfully called SNPs in TCS were checked for read depth (DP), mapping quality (MQ), and phred-scaled quality (QUAL) on each chromosome. The problematic regions were identified by observation, and reported regions started from the first SNP of MQ < 50. Three distinct factors led to the identification of the problematic SNPs: low concordance with Versa50K in genotyping, interquartile range (IQR) outliers for sequencing depth and SNPs not captured in the centre of the target regions. The SNPs of the outliers with extremely high or low coverages were identified using the IQR method, i.e., those outside of 1.5X IQR [48]. The sequencing depth on the SNP (depth_snp_) and the average sequencing depth of the target region (depth_ave_) were calculated separately. The target region was defined as ~250 bases surrounding the SNP at the centre. The SNPs with depth_snp_ < depth_ave_ were judged not captured in the middle of the target region. We categorised the SNPs with the aforementioned issues as problematic SNPs, listed them in a table with their MI and MAF, and marked if they were the common SNPs from both BCFtools and GATK methods.

### 2.7. Genomic Relationship Matrix Construction

The genotypes of the SNP array and TCS were imputed separately using minimac4 [49] with a reference panel (*n* = 417) from the same Wagyu population. The genomic relationship matrices (GRMs) were computed with the VanRaden method using the imputed genotypes [50]. The Pearson correlation coefficient [51] was used to check the correlation between the matrixes built from the genotypes from TCS and the SNP array.

### 2.8. Genotype of Interest for Commercial Traits

As the animals chosen for this study were Wagyu, we tested for the genotypes of five key breeding markers. The markers of 56 animals for traits, including Band 3 Spherocytosis, Factor XI Deficiency, Fading Calf Syndrome, Claudin 16 Deficiency and Chediak-Higashi Syndrome (Table S4), were collected in the format of counting the B allele (i.e., 0, 1 and 2) for TCS and SNP data. The concordance of the genotyping results for the markers was checked.

### 2.9. Sex Assignment with Markers from Sex Chromosomes

The probes were designed to capture five and 2566 SNPs located on the Y and X chromosome, respectively. For sex assignment analysis, the pseudoautosomal region (PAR) [52] was excluded. By identifying the presence or absence of Y-specific SNPs and analysis of the heterozygosity level of the non-PAR region of X chromosome, it was possible to assign the sex of the animals. 

### 2.10. Down-Sampling of the TCS Data to 30X

It has been suggested by TWIST that the commercial applications of TCS may use the sequencing depth of 30X per probe (personal communication). To evaluate this scenario, we down-sampled the TCS data to 30X for the 56 samples, and then, re-analysed the concordance, R^2^, parentage, sex prediction, genotype of interest and GRM using the down-sampled data.

## 3. Results

### 3.1. Descriptive Statistics and Mapping Quality

**Figure 1:**
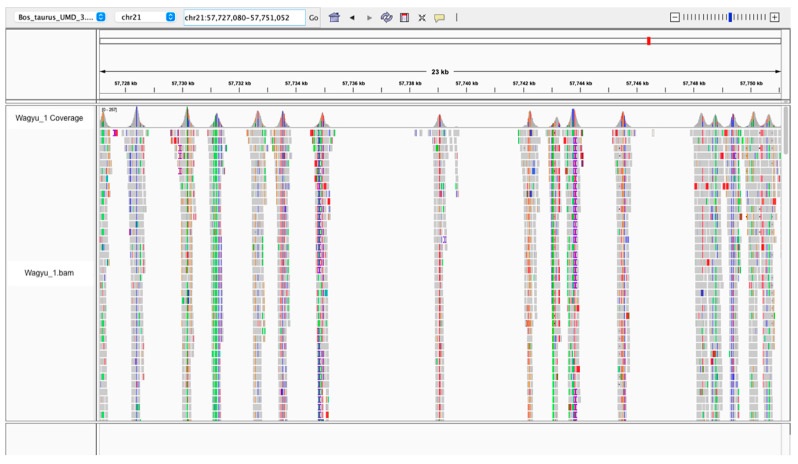
Sequencing depths of the targeted sites were confirmed through manual inspection with IGV. An example region showing read depth in the targeted regions of ~250 bps centred at the chosen SNP. The colors indicate read mismatches with the reference.

**Figure 2:**
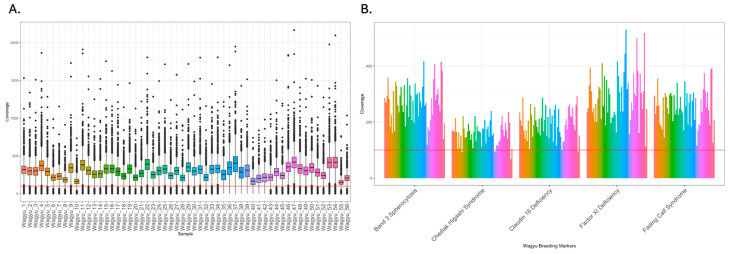
The coverage of targeted SNPs and the location of five important Wagyu markers. (**A**) Boxplot of the coverage of sample-wise targeted SNPs. The X-axis denotes the sample ID, with the Y-axis denoting the sequencing depth at the targeted SNP positions. The red line denotes 100X coverage. (**B**) The sequencing depth of five genotype of interest of 56 Wagyu samples. The red line denotes 100X coverage of the targeted regions. Each different coloured bar represents a sample, and the identity of the sample follows panel A.

The sequencing generated an average of 33.91 million raw reads across the 56 samples. Following the completion of quality control and data cleaning procedures, the average number of cleaned reads was 32.66 million (Table S5). The average alignment rate of these reads to the reference genome was 99.96%. Cursory checks of targeted regions with Integrative Genomics Viewer (IGV) showed the expected pattern of sequencing coverage, i.e., normal distribution centred at the chosen SNP (Figure 1). Only 1.3% of SNPs (n = 819) did not reach the intended sequencing depth of 100X. The median sequencing depths of SNPs of all samples were above the target sequencing depth of 100X (Table S6, Figure 2). The overall coverage across the genome was equivalent to ~1X per sample. 

Overall, the average depth per SNP was ~260X, and the majority of them were between 100X and 500X (Figure 2A). Using the interquartile range (IQR) method, 2477 SNPs were identified as outliers based on their sequencing depths and were separately flagged as having either low (n = 2381) or high coverage (n = 96). Only 156 SNPs were not positioned at the centre in the targeted regions. The sequencing depths of the five key markers of the genetic disorders were on average higher than 100X (Figure 2B).

### 3.2. Preference for Using BCFtools Instead of GATK

The genotypes obtained by BCFtools had high concordance (>95%) with the Versa50K SNP array (Figure S2). In contrast, GATK resulted in a higher number of SNPs but had slightly lower concordance (>92%), particularly in relation to the X chromosome (Figure S3). The concordance of GATK remained the same with or without the base call recalibration. BCFtools used less storage space for SNP calling in comparison to GATK. Given the higher concordance of BCFtools and the lower usage of storage space for SNP calling, the remaining analysis was based on BCFtools results. 

### 3.3. High Concordance between TCS and Versa50K

Out of the 71,746 targeted variants, 71,553 SNPs and 166 indels were successfully identified (Table S7). There were 68,912 SNPs successfully called across all samples. On average, 618 SNPs failed to be called in each sample (Table S8) and 2641 SNPs failed for at least one sample. After the removal of 173 SNPs with an MI higher than 5% (Table S9), 37,130 common SNPs could be compared between the TCS and Versa50K SNP array. Two samples had call rates below 90% in the SNP array data and they were dropped from further analysis. 

**Figure 3:**
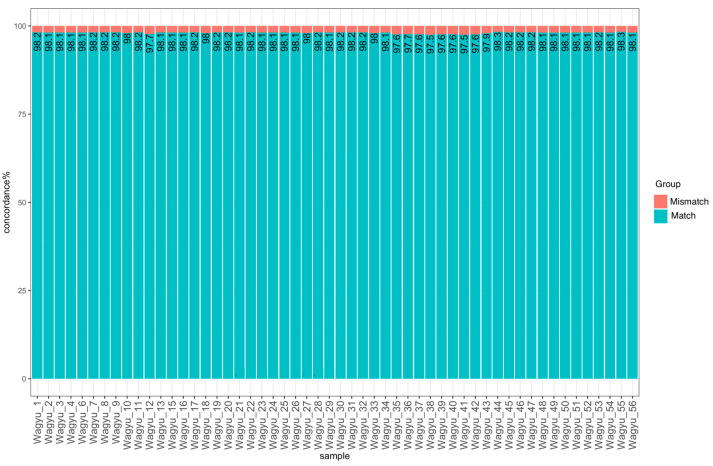
The concordance of genotypes between TCS and Versa50K SNP array for 54 samples after the removal of low-quality SNPs and samples.

The mean concordance, R^2^ (coefficient of determination), and Cohen kappa coefficient across all samples were 98% (Figure 3), 0.98 and 0.97, respectively. The Cohen kappa coefficient for individual samples is provided in Table S10. These results were obtained after excluding the SNPs with a high MI and two samples (Wagyu_5 and Wagyu_14) with less than 90% call rates in the SNP array from the analysis (Figures S4 and S5).

A total of 99 targeted SNPs were absent in all the samples (Figure S6). A large proportion of the SNPs (24,801, 66.8%) was concordant for all 56 samples. Another 15.1% (5592) of the SNPs were concordant between two technologies throughout for 55 samples, and 5.7% (2101) of the SNPs were consistent across 54 samples. Only around 2% of the SNPs were concordant for less than five samples. The R^2^ on SNP-wise analysis supported the high agreement between TCS and SNP array (Table S11).

### 3.4. Problematic Regions in TCS

**Figure 4:**
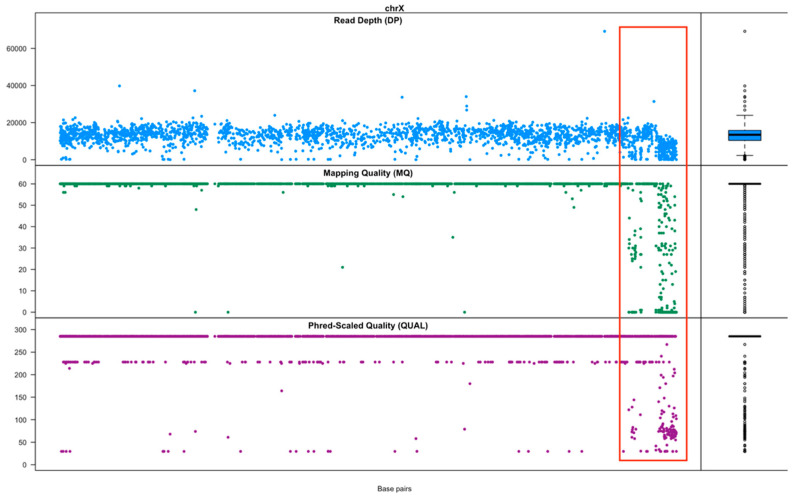
Distribution of SNPs on the X chromosome and the read depth (DP), mapping quality (MQ), and phred-scaled quality (QUAL). DP refers to the overall read depth from all 56 samples supporting the genotype call. MQ refers to the root mean square mapping quality of all the reads spanning the given variant site. QUAL can vary from 0 to infinity, and a higher score means a greater likelihood that a genotype is accurate. The red box denotes problematic regions.

The chromosome-wise distribution of the called SNPs in TCS, along with their read depth (DP), mapping quality (MQ) and phred-scaled quality (QUAL), are shown in Figure S7. These values were used to identify the regions of concern. For instance, DP, MQ and QUAL all exhibited a substantial reduction towards the end of the X chromosome q-arm. The accuracy of genotypes from the SNP at position 131,370,676 to the end of the X chromosome was likely compromised (Figure 4). The concordance of genotypes located in these regions between the two technologies were relatively low. Similarly, a low mapping rate was discovered using a targeted SNP at position 24,093,567 on the Y chromosome. In addition, there are sections in the middle of some chromosomes, specifically chr2:104,825,968–105,006,704, chr12:70,670,517–75,924,170 and chr27:5,194,237–5,919,047, which also showed a decline in mapping quality. A comprehensive summary of the chromosome-specific details pertaining to the successfully called SNPs is shown in Table S12, including the chromosome average MAF and the mean inter probes spacing.

### 3.5. Problematic SNPs in TCS

**Figure 5:**
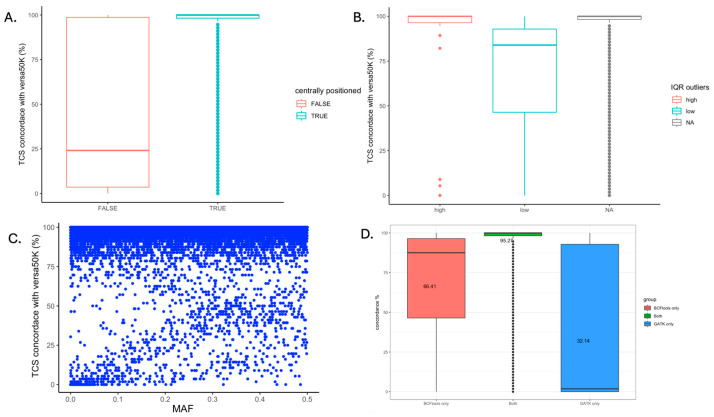
Analysis of problematic SNPs due to the non-central position, coverage outlier, MAF and overlap of SNP calling tools. (**A**) The Y-axis presents the concordance of genotypes between TCS and the Versa50K SNP array. The SNPs were grouped by whether they were centrally positioned in the target regions. (**B**) The Y-axis presents the concordance of the genotypes between TCS and the Versa50K SNP array. The SNPs were groups by different types of interquartile range (IQR) outliers with “high” for high sequencing depth and “low” for low sequencing depth, and “NA” are not outliers by IQR definition. (**C**) The concordance of genotypes for SNPs between TCS and the Versa50K SNP array was plotted with their minor allele frequencies (MAFs). MAFs were calculated using the Versa50K SNP array data. (**D**) The concordance of genotypes between TCS and the Versa50K SNP array were grouped by SNP calling methods. There are 37,240 genotypes called by both BCFtools and GATK, 63 genotypes called by BCFtools only and 1816 genotypes called by GATK only.

The results indicated that SNPs located at the centre of the probes (Figure 5A), excluding outliers in terms of sequencing depths, showed (Figure 5B) higher concordance between TCS and the SNP array. In addition, the TCS concordance and minor allele frequencies are shown in Figure 5C, which indicated the expected outcome, that detection of genotype was not strongly influenced by allele frequency. A list of 3,132 problematic SNPs was reported based on low concordance, outlier of sequencing depths and non-central SNP position (Table S13). The list included a summary of 843 SNPs with a concordance of less than or equal to 50%, 2,381 IQR outliers with a low sequencing depth, 96 IQR outliers with a high sequencing depth and 156 SNPs not located at the centre of the target regions. The locations of these problematic SNPs are shown in Figure S8, which notably overlap with problematic regions from X:131,370,676 until the end of the X chromosome. Beyond the previously mentioned reasons for problematic SNPs, we found that SNPs not situated in the centre of the target regions were more prone to lower concordance (Figure 5A). Moreover, ~95% of the SNPs called were common between BCFtools and GATK, and these SNPs showed higher concordance with the SNP array when compared to SNPs called by only one tool (Figure 5D).

### 3.6. GRM between TCS and SNP Array Is Highly Correlated

The Pearson correlation coefficient was calculated between the corresponding elements of the genomic relationship matrices (GRMs) derived from TCS and SNP array genotypes. Overall, there was a 0.9998 correlation between the GRMs computed using genotypes from the two technologies on the same sample (Figure S9). 

### 3.7. High Concordance of the Genotype of Interest

All five Wagyu breeding specific markers were 100% concordant between TCS and the SNP array. However, as the marker for Factor XI was a 15 bp indels, the GATK method was preferred as the method to call this indel instead of BCFtools. Additionally, GATK gave a higher concordance with the SNP array than BCFtools. 

### 3.8. Accurate Sex Assignment and Parentage

Three SNPs were successfully targeted on the Y chromosome, and two of these SNPs had 100% concordance with the SNP array. The sexes assigned by TCS data and SNP array data, which utilised the heterozygosity level in non-PAR X chromosome in addition to the presence or absence of Y markers, were 100% concordant with each other (Table S14). The predicted parents of the 56 samples also matched parents predicted from SNP array 100%. The database used to search for parents was based on SNP array genotypes of the same Wagyu population. 

### 3.9. Down-Sampled 30X TCS Is Still Highly Concordant with the SNP Array

After down-sampling, the distribution of the average sequencing depth for SNPs are shown in Figure S10. As shown, the distribution of the sequencing depth centred at around 30X. The concordance for the down-sampled data was, on average, 1% lower than the original comparison with 100X more coverage (Figure S11). The five breeding markers, sex assignment and parentage were 100% consistent with the high coverage. The GRMs showed a correlation of ~1 between the 30X and the original coverage.

## 4. Discussion

The objective of the project was to assess whether TCS was a suitable alternative to SNP array genotyping in cattle. The overall genotypes obtained from the TCS were highly concordant (~98%) with the SNP array, and the SNPs between the two technologies were highly correlated (R^2^ = 0.98). Although the concordances were slightly different for two batches, the difference in coverage and concordance were likely due to the differences in the batch rather than sex. The average sample call rates for the ~70K SNPs targeted was 98.8%. Even when the TCS data were down-sampled to 30X coverage, the concordance between the TCS and the SNP array remained high (~97%). Our results are in agreement with a previous study that developed a TCS panel of ~110K SNPs for Chinese cattle and reported 98.17% concordance between their TCS and the Illumina Bovine HD BeadChip [7]. In human whole-exome sequencing (WES), which is a similar technology to TCS, a high concordance of 99.99% with the SNP array was observed [53]. Theoretically, there is no upper limit to the number of probes that can be applied for hybridization capture. A study used TWIST probes to target 1.24 million SNPs in ancient human samples [54]. From the SNP probe design to synthesis, the whole process can be completed in less than a month, which makes TCS a flexible alternative to an SNP array. TCS is a suitable replacement for an SNP array since their concordance and call rates are both very high, and the technology is capable of targeting millions of SNPs with short design and deployment timelines. However, WGS may be preferred in situations where several million SNPs are required, as probe synthesis and more complex sequencing library preparation for TCS can become costly at this scale. Moreover, WGS discovers SNPs in an unbiased manner and does not require a priori information of the genomic sequence for probe design. The choice between TCS and WGS depends on the study’s specific requirements, including the number of target SNPs, desired flexibility and budget constraints. Both TCS and WGS are sequencing-based methods and utilise similar bioinformatics tools for genotyping, which differentiates them from traditional SNP arrays. In this context, TCS serves as a transitional technology towards the potential wider adoption of WGS for routine genotyping.

The key distinction between our cattle panel and the Chinese 110K SNP panel [7] lies in our SNP design approach. We based our selection on the overlap of two medium-density Illumina SNP arrays (Versa50K and GGPLD30K), which are routinely used for genotyping the Wagyu cattle in our research. This strategy ensures compatibility with our existing genomic data and research practices. The Chinese 110K SNP panel was based on the high-density Bovine BeadChip and other annotation such as SNPs uncovered by GWAS, eQTL, the assay for transposase-accessible chromatin with sequencing (ATAC-seq) and other information. However, it was designed for use with Huaxi cattle and other Chinese breeds. We showed that our TCS 70K SNP panel produced the same results of parentage, sex prediction and five Wagyu genotype-of-interest markers when compared to SNP array. The GRM produced by our TCS was also highly correlated (close to unity) with the one by an SNP array. The Chinese 110K SNP panel has been reported to show accurate genomic economic breeding values and could be used in GWAS [7]. These results demonstrated that TCS is a genotyping solution for animal breeding applications. 

A popular alternative to TCS is low-pass WGS, which involves sequencing the genome in an unbiased manner, but at a low coverage of sequencing, typically approximately 1X coverage. This requires applying genotype imputation to fill in missing genotypes. In cattle, a study that utilised 1X low-pass WGS has demonstrated high average concordance rates of 99.6% and 98.8% for Holstein and Simmental breeds, respectively [55]. These authors created a publicly available SNP reference panel of 2,976 cattle to facilitate the genotype imputation process. In comparison with TCS, there are two main differences of low-pass WGS. One is the fact that imputation is required for low-pass WGS, and hence the accuracy of certain genotypes is lower than TCS that does not require imputation. For certain genotype-of-interest markers that are critical for breeding, such as the five Wagyu breeding markers of this study, ideally no imputation should be performed to obtain accurate genotypes. Another main difference is that low-pass WGS will likely discover more SNPs than TCS, without the need for a more complex sequencing library preparation step that requires probes. In terms of bioinformatics requirements, the storage size of the sequencing information is similar between low-pass WGS and TCS, but low-pass WGS will likely require more computing time due to the imputation step. Moreover, the imputation panel will need to be updated with new animals perhaps every 2 years to capture relevant haplotypes as animals are being continually selected for genetic improvement.

The TWIST SNP probes were designed to be 120 bases in length, and these probes capture up to 250 bases of sequences around the targeted SNPs. Even with sequence mismatches >10% difference between the probes and targeted sequence in the genome, TCS will still work effectively, as we have found with one of the Wagyu breeding markers, Factor XI deficiency. The Factor XI deficiency variant in Wagyu was initially described as a 15 bp insertion in exon 9 of F11 and an SNP (C>A) at the insertion site at chr27:15,362,363 (UMD3.1.1) [56]. In SNP array technology, the indel and surrounding sequences of F11 are not seen, but only the SNP at chr27:15,362,363 is reported, which functions as a proxy to the 15 bp indel status via the linkage disequilibrium. Since TCS produces ~250 bases around the SNP of interest, there are likely many other SNPs and indels that can be found within the captured region. Some researchers have used the term, multiple SNPs (mSNPs) [57,58] to describe SNPs that have been found alongside the targeted SNPs. These mSNPs can be useful to define haplotypes around the target loci. Some researchers have reported improved accuracies of genomic selection of growth and reproductive traits by 2 to 6% in pigs using mSNPs [57]. In maize, it has been reported that the use of mSNPs is better for genetic diversity detection, linkage disequilibrium decay analysis and GWAS [57]. In our study, although mSNPs are likely present, our analysis pipeline focused solely on targeted SNPs, as our primary objective was to evaluate the efficacy of TCS as a genotyping solution.

There are other aspects of TCS that will be useful to explore in the future. One is the use of less sequencing coverage such as the range from 5X to 10X coverage, and test how well imputation produces accurate genotypes in comparison to the 30X or 100X coverage of TCS. This has the potential to reduce sequencing cost; however, the use of imputation will increase computing requirements and likely be less accurate than the high-coverage sequencing of TCS. Future TCS applications should explore enhanced panel design by incorporating diverse genetic markers. These could include eQTLs, ChIP-seq data from potential enhancer regions, conserved genomic sequences and other informative sources that may indicate potential causal mutations for traits of interest [59]. This approach would likely increase the power of the panel to detect meaningful genetic variations.

## 5. Conclusions

In summary, TCS emerges as a compelling alternative to SNP arrays for animal breeding applications, offering comparable accuracy with high concordance and reliable genotype call rates. TCS provides flexibility in targeting numerous SNPs, coupled with rapid probe synthesis and sequencing turnaround. Unlike low-pass WGS, TCS eliminates the need for genotype imputation, making it particularly advantageous for an accurate genotyping of specific markers in genomic selection applications. Future investigations should explore optimising lower-coverage TCS with imputation to save costs, and design targeted SNPs based on functional information from the literature.

## Data Availability

Data may be available upon request.

## Supplementary Materials

The following supporting information can be downloaded at: https://www.mdpi.com/article/10.3390/genes15091218/s1, Figure S1. The bioinformatics pipeline. The key steps for analysing probe-captured data are shown in green. Orange indicates the steps for analysing SNP array data. The grey blocks show the software versions and comments. The block in lighter blue shows the step until which the concordance has been compared between technologies. Figure S2. The concordance of genotypes for Versa50K and TCS using either BCFtools or GATK for genotype calling. The blue colour shows the percentage of SNPs that are concordant with Versa50K, whereas the red colour shows the percentage of SNPs that are inconsistent between the two technologies. Grey colour shows the percentage of SNPs that were not called using different methods. The red reference line was drawn at 95% of the concordance. Figure S3. The concordance of SNPs called by GATK and BCFtools on each chromosome. The genotyping concordance between the target captured sequencing and SNP array for SNPs called on different chromosomes. Y chromosomes with only three SNPs are not listed. The concordance of SNPs on the Y chromosome can be found in Table S11. Figure S4. Comparison of the call rates of TCS and Versa50K at the sample level using 37,130 SNPs that are commonly detected. TCS has a significantly higher (*t*-test: *p*-value = 1.309 × 10^−2^) call rate than Versa50K, with a mean call rate of 0.991. Samples Wagyu_5, Wagyu_14, Wagyu_12, Wagyu_18, Wagyu_2 and Wagyu_43 are outliers, with a low call rate in Versa50K, whereas the mean call rate is 0.983. Figure S5. The concordance of genotypes between TCS and Versa50K for 56 samples in percentage for 37,130 SNPs. The light blue bar shows the percentage of concordant SNPs in each sample, whereas the red bar shows the discordant SNPs. The percentage of SNPs that cannot be compared due to no call (either in TCS or Versa50K) is shown in grey. Figure S6. The distribution of SNPs that match or fail in the number of samples between the two technologies. The maximum of the Y-axis is 37,130 and the maximum of the X-axis is 56 samples, since the bin size is 1. More than half of the SNPs (24,801, 66.80%) are concordant between the two technologies for all 56 samples. The red arrow in the diagram indicates the 99 SNPs that were not successfully identified in any of the samples during the TCS analysis. The red arrow in the figure pointed the SNPs that were not successfully identified in any of the samples during the TCS analysis. Figure S7. The distribution of 71,553 single-nucleotide polymorphisms (SNPs) in TCS, categorised by chromosomes. This includes 71,746 targeted SNPs, with 193 SNPs excluded due to not being called in any sample. The corresponding read depth (DP), mapping quality (MQ) and phred-scaled quality (QUAL) are displayed. Figure S8. The location of the problematic SNPs listed in Table S13 on each chromosome. Figure S9. The scatter plot of the same positions in the genomic relationship matrix (GRM) built either with genotypes from the SNP array or targeted captured sequencing (TCS). The dots are highly clustered around the blue line means the two GRMs are very similar with differences ranging from −0.0065 to 0.0168. Figure S10. The distribution of average sequencing depth for 71,553 SNPs crossing 54 samples (two samples were removed as low quality for Versa50K) after down-sampling for 30X. Figure S11. The concordance of genotypes between TCS and the SNP array for average sequencing depths of 260X and 30X The figure was coloured for 54 samples. Table S1. The data yield after ligation for the blood samples 56 animals. Table S2. The call rate of SNP array (Vers50K) for the 56 selected Wagyu cattle. Table S3. The 332 trios from the same population as the test animals used for the Mendelian inconsistency (MI) check. The progeny and their parents are listed in the table. Number of SNPs tested and failed on sample-wise analysis were summarised. The percentage of SNPs selected as the MI are listed in the column trio_MI. Table S4. The quality control states of the target capture sequencing on sample-wise analysis. The number of raw and clean reads are shown in millions. The mapping rate and duplication rate are shown in percentage, along with the sample wise average coverage after cleaning. Table S5. The list of targeted SNPs with their coverage for each sample. The mean coverage of the target SNPs and probes were listed separately in the columns of snp_mean and probe_mean. The column snp_centrally_positioned is marking if this SNP is centrally located in the middle of the target regions. The interquartile range (IQR) outliers with a high or low coverage were marked for those SNPs. Table S6. Cattle commercial marks of traits were studied in the paper. Eighteen marks, along with their correlated traits, locations, causative allele and alleles, are listed in the table. Table S7. The list of the called indels and 28 SNPs failed in genotype calling. Either indel or reasons of failing were recorded in the Type column. The ref. and alt. allele at the positions are given with their phred-scaled quality (QUAL) values. The genotypes of those indels for each sample as shown in the table. Table S8. The calling status of the targeted SNPs. Fifty-six samples of Wagyu cattle were used for target-captured sequencing, and the average call rate is 0.989 for those SNPs. Table S9. The SNPs tested for Mendelian inconsistency (MI). The IDs of the SNPs on Versa50K and their locations are listed in the table. The call rate and minor allele frequency (MAF) calculated with SNP array data are listed. The number of trios used for testing (n_trio) the number of the trio detected for MI (n_error) and either percentage (trio_MI) are calculated. Table S10. The sample-wised Cohen kappa coefficient values. Wagyu_5 and Wagyu_14 with call rates lower than 90% of the SNP array were removed. Table S11. A list of genotypes compared for concordance and R^2^ between target-captured sequencing and the SNP array. In total, the genotypes of 37,130 were compared between the technologies. The matching, non-matching and failed results for different technologies are listed in the columns match_w_snpchip, nomatch_w_snpchip, n_sample_failed_CHIP and n_sample_failed_TCS separately. The R^2^ for each SNP were calculated using 56 Wagyu cattle samples. Table S12. The number of called SNPs, mean minor allele frequency (MAF) and probe spacing of chromosome-wise analysis. Table S13. A list of all problematic SNPs detected from target-captured sequencing. The problems can be categorised into different types: first, outliers (either low or high) at a sequencing depth that have been identified by the interquartile range (IQR) method; second, the SNPs not located at the middle of the target regions; third, the SNPs have low concordance between target-captured sequencing and the SNP array (Versa50K). The percentage of Mendelian inconsistency calculated with SNP array data with 332 trio from the same population are collected for SNPs in the column trio_MI. The minor allele frequency (MAF) calculated with SNP array data are listed and grouped into >0.1 and <=0.1 in column Mark_maf. Table S14. The comparison of assigned sex using target captured sequencing (TCS) data and SNP array. The predicted X column showing the number of X chromosomes predicted. The predicted Y column showing the number of Y chromosomes predicted. The assigned sex for TCS and SNP array are listed in the column assigned sex TCS and sex SNP array.
