# Supplementary material for: Evaluating the Efficacy of Target Capture Sequencing for Genotyping in Cattle
Source: Genes (Basel). 2024 Sep 18;15(9):1218. doi: 10.3390/genes15091218 (PMC11431841; doi:10.3390/genes15091218)
Supplement: Supplementary file 1 [file genes-15-01218-s001.zip › Probe_capture_paper_supplementary_files_20240910/Sub_Figures/FigureS6_Num_of_cor_failed.docx]

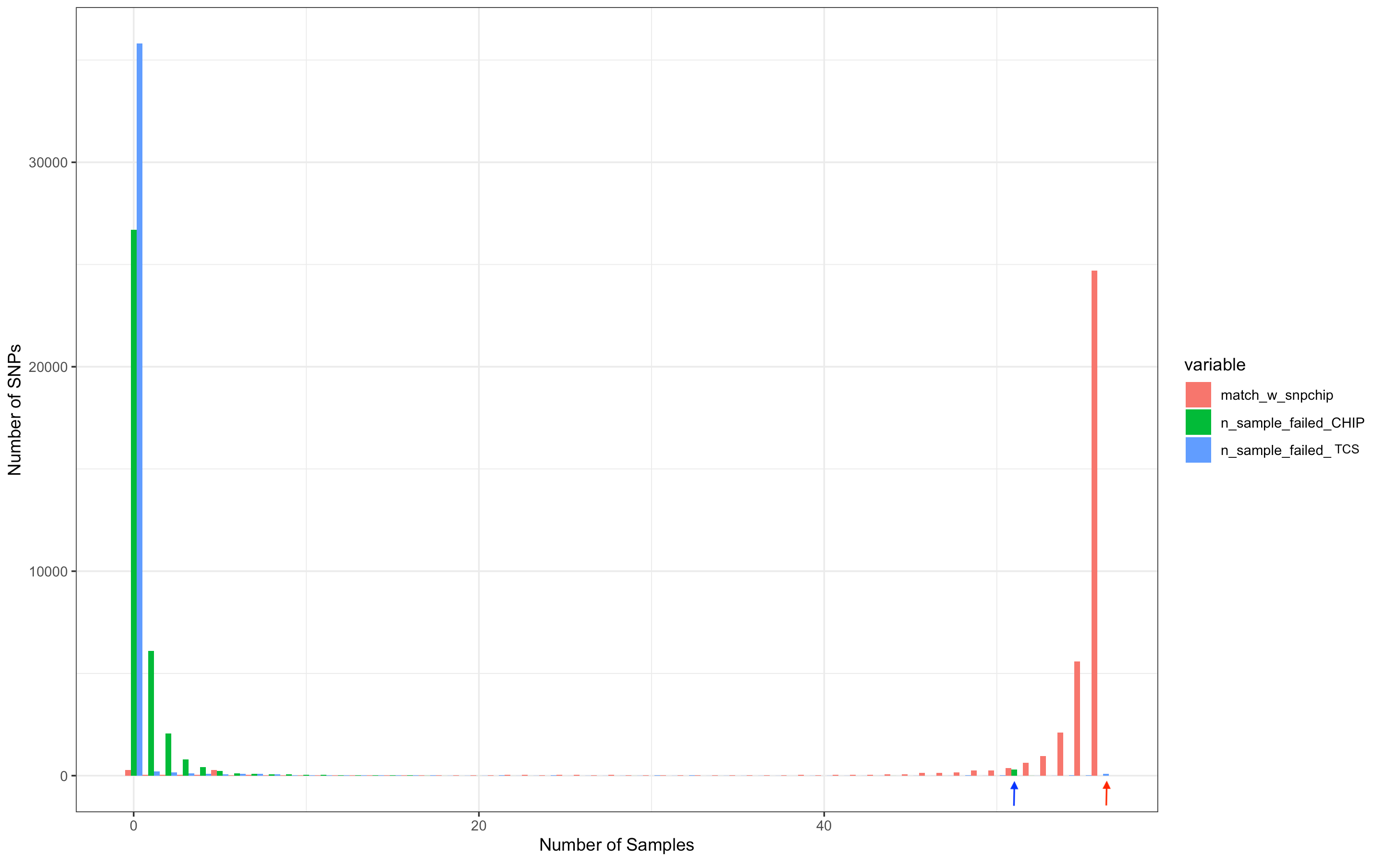


**Figure S6. The distribution of SNPs that match or fail in the number of samples between two technologies. The** maximum of the Y axis is 37130, the maximum of the X axis is 54 samples since the bin size is 1. More than half of the SNPs (24801, 66.80%) are concordant between two technologies for all 54 samples. The red arrow in the diagram indicates the 99 SNPs that were not successfully identified in any of the samples during the TCS analysis. The red arrow in the figure pointed the SNPs that were not successfully identified in any of the samples during the TCS analysis.
