# Supplementary material for: Evaluating the Efficacy of Target Capture Sequencing for Genotyping in Cattle
Source: Genes (Basel). 2024 Sep 18;15(9):1218. doi: 10.3390/genes15091218 (PMC11431841; doi:10.3390/genes15091218)
Supplement: Supplementary file 1 [file genes-15-01218-s001.zip › Probe_capture_paper_supplementary_files_20240910/Sub_Figures/FigureS9_Compare_GRM.docx]

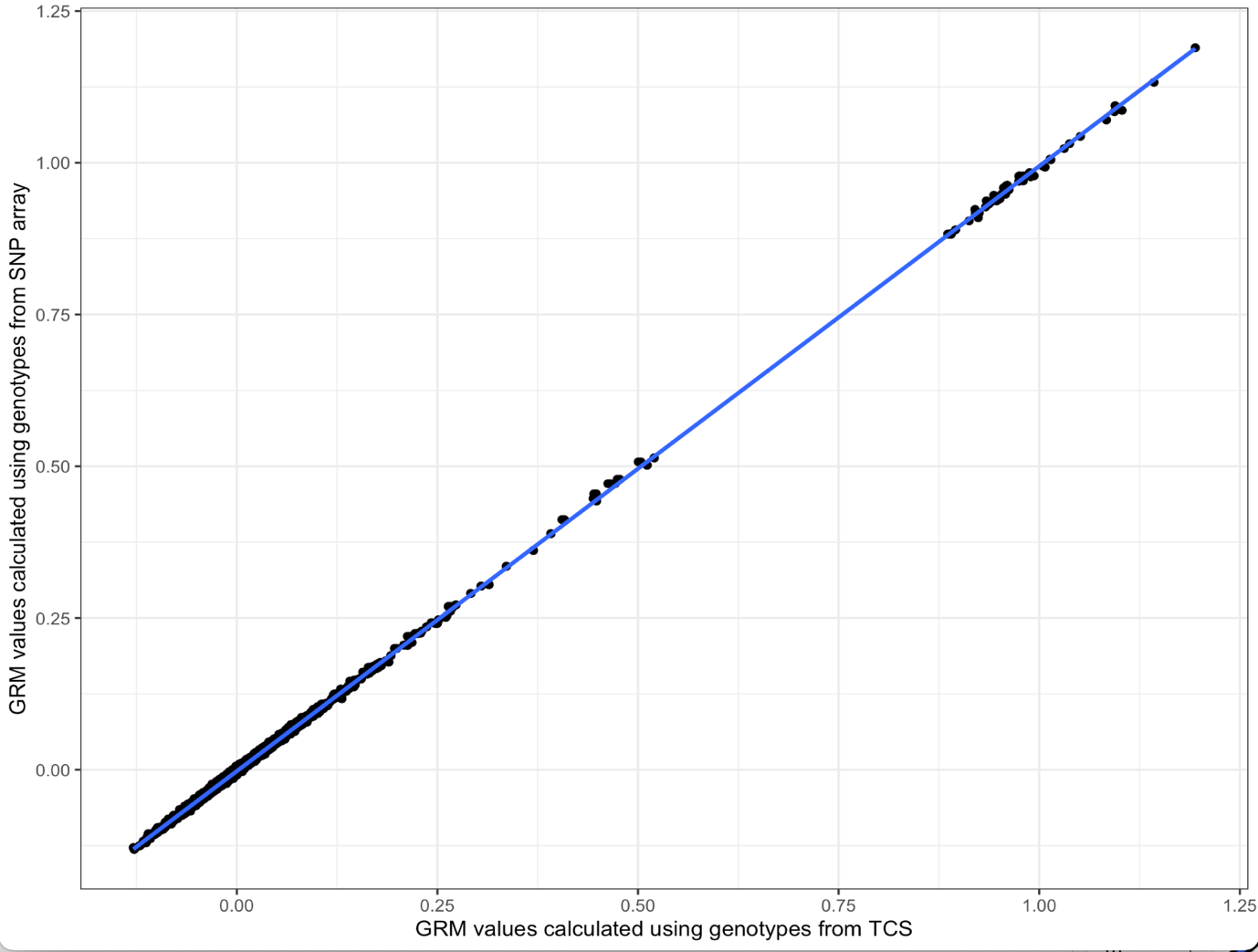


**Figure S9. The scatter plot of the same positions in the Genomic relationship matrix (GRM) built either with genotypes from SNP array or targeted captured sequencing (TCS).** The dots are highly clustered around the blue line means the two GRMs are very similar with differences ranging from -0.0065 to 0.0168.
