# Supplementary material for: Evaluating the Efficacy of Target Capture Sequencing for Genotyping in Cattle
Source: Genes (Basel). 2024 Sep 18;15(9):1218. doi: 10.3390/genes15091218 (PMC11431841; doi:10.3390/genes15091218)
Supplement: Supplementary file 1 [file genes-15-01218-s001.zip › Probe_capture_paper_supplementary_files_20240910/Sub_Figures/FigureS5_Sum_concordance_Versa50K_56_37130.docx]

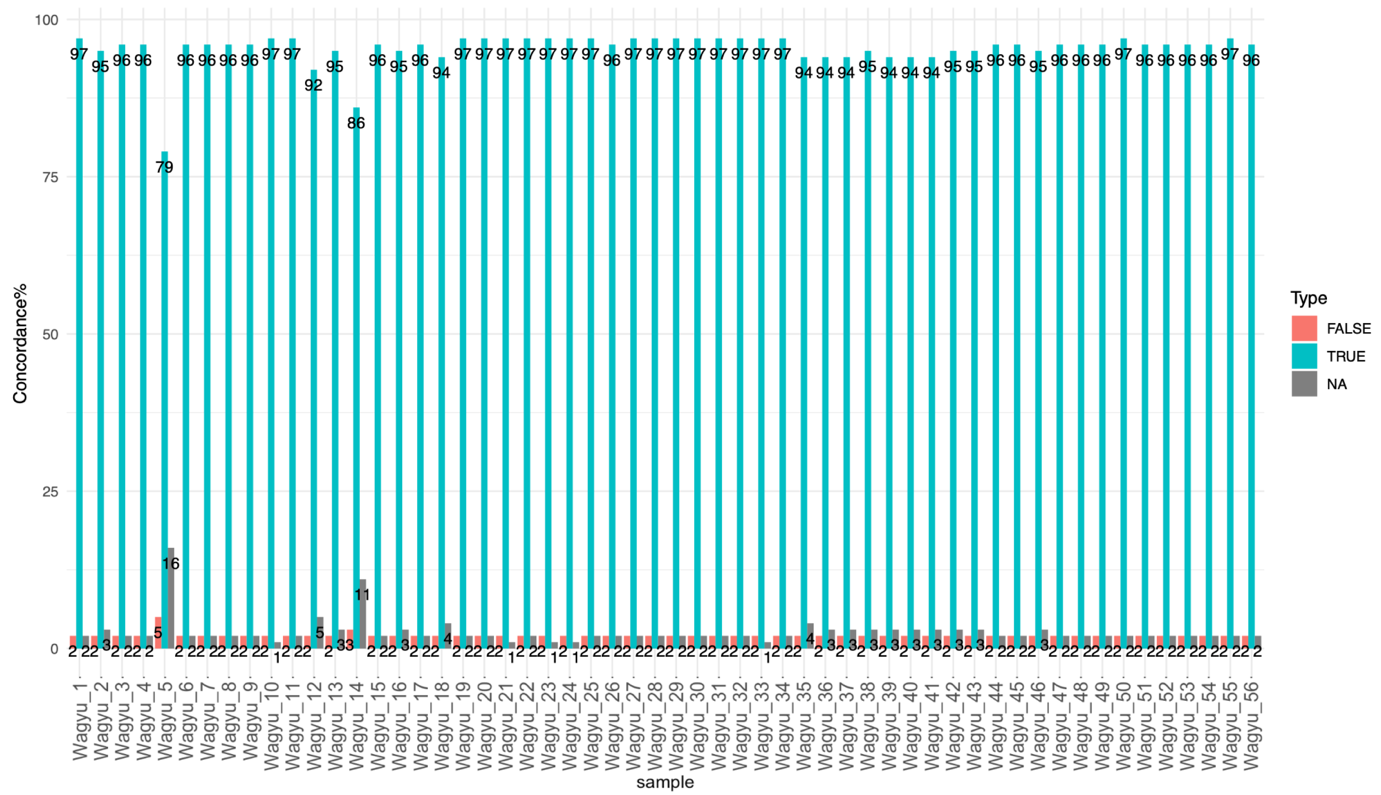


**Figure S5. The concordance of genotypes between TCS and Versa50K for 56 samples in percentage for 37130 SNPs.** The light blue bar shows the percentage of concordant SNPs in each sample, whereas the red bar shows the SNPs of miss-concordant. The percentage of SNPs that cannot be compared due to no call (either in TCS or Versa50K) is shown in grey.
