# Supplementary material for: Evaluating the Efficacy of Target Capture Sequencing for Genotyping in Cattle
Source: Genes (Basel). 2024 Sep 18;15(9):1218. doi: 10.3390/genes15091218 (PMC11431841; doi:10.3390/genes15091218)
Supplement: Supplementary file 1 [file genes-15-01218-s001.zip › Probe_capture_paper_supplementary_files_20240910/Sub_Figures/FigureS1_bioinformatic_pipeline.docx]

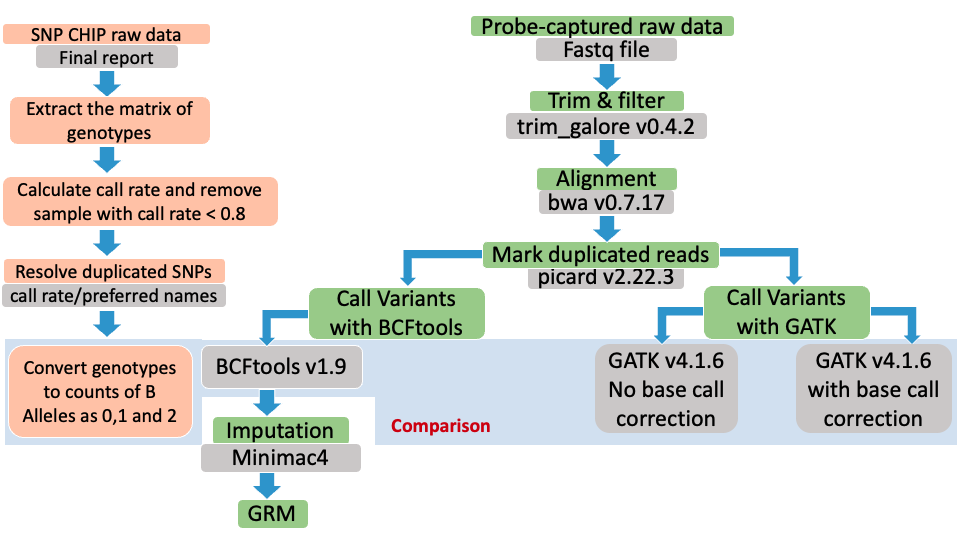


**Figure S1. The bioinformatic pipeline.** The key steps for analyzing probe-captured data are shown in green. Orange indicates the steps for analyzing SNP array data. The grey blocks show the software versions and comments. The block in lighter bule is showing until which step the concordance has been compared between the technologies.
