# Supplementary material for: Evaluating the Efficacy of Target Capture Sequencing for Genotyping in Cattle
Source: Genes (Basel). 2024 Sep 18;15(9):1218. doi: 10.3390/genes15091218 (PMC11431841; doi:10.3390/genes15091218)
Supplement: Supplementary file 1 [file genes-15-01218-s001.zip › Probe_capture_paper_supplementary_files_20240910/Sub_Figures/FigureS2_GATK_BCFTOOLS.docx]

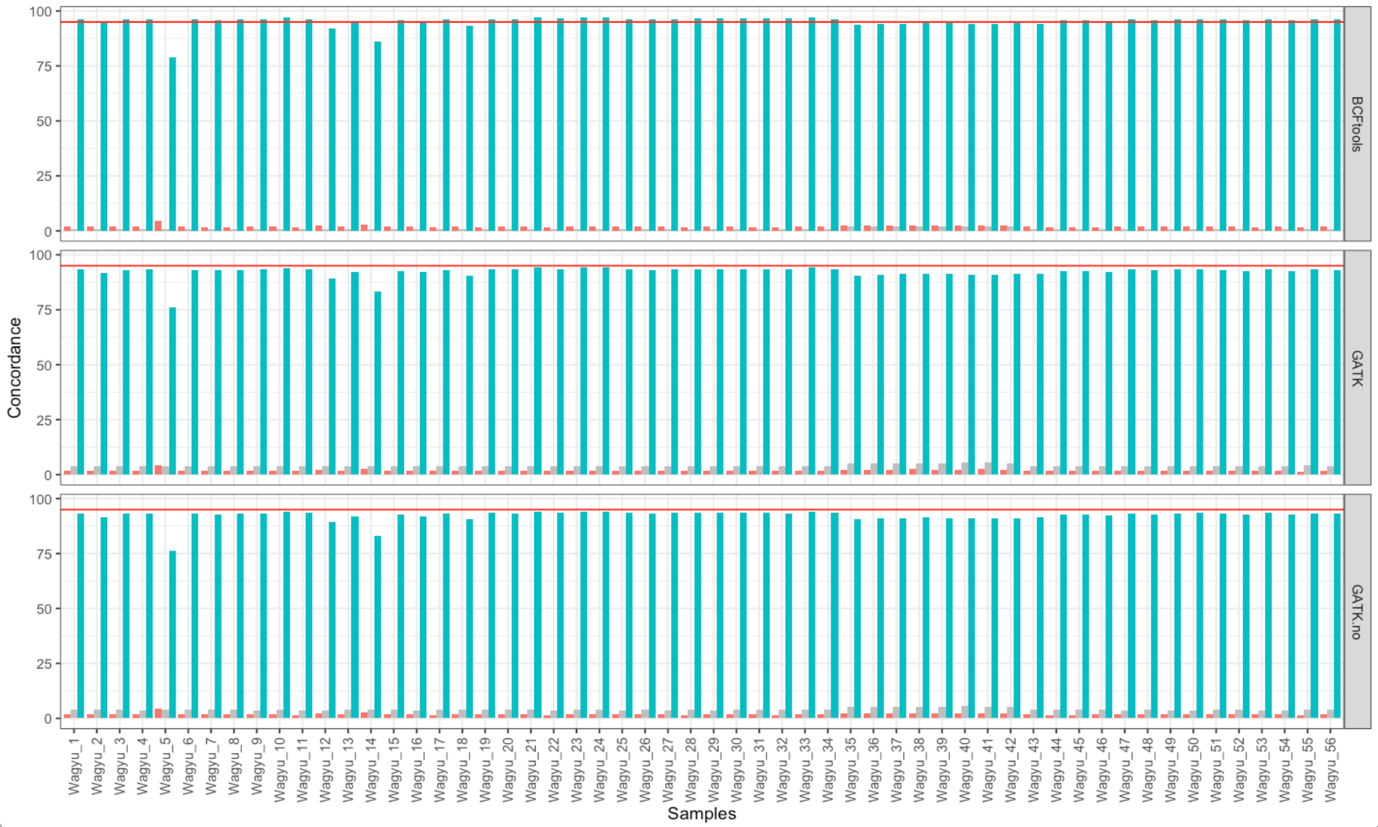


**Figure S2. The concordance of genotypes for Versa50K and TCS using either BCFtools or GATK for genotype calling.** The blue color shows the percentage of SNPs that are concordant with Versa50K, whereas the red color shows the percentage of SNPs that are inconsistent between the two technologies. Grey color shows the percentage of SNPs that were not called using different methods. The red reference line was drawn at 95% of the concordance.
