# Supplementary material for: Evaluating the Efficacy of Target Capture Sequencing for Genotyping in Cattle
Source: Genes (Basel). 2024 Sep 18;15(9):1218. doi: 10.3390/genes15091218 (PMC11431841; doi:10.3390/genes15091218)
Supplement: Supplementary file 1 [file genes-15-01218-s001.zip › Probe_capture_paper_supplementary_files_20240910/Sub_Figures/FigureS7_Read_distribution_chr.docx]

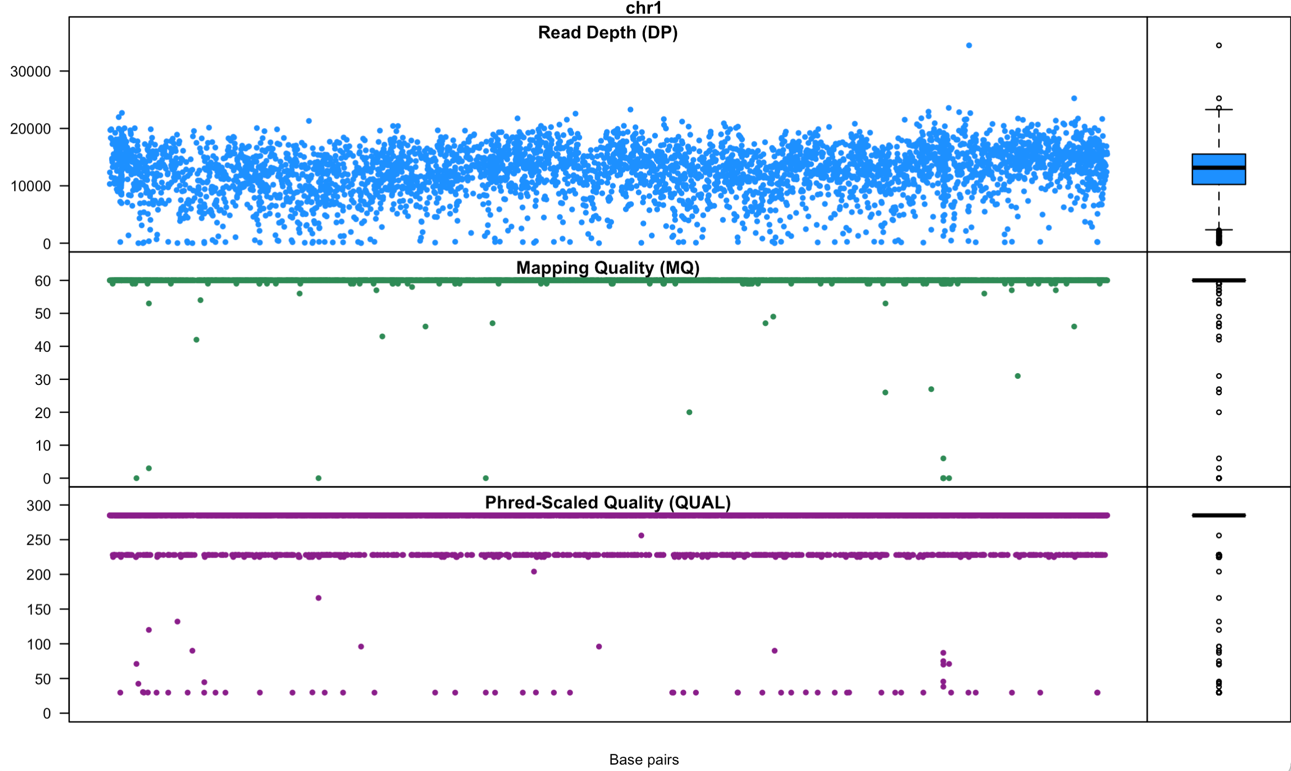


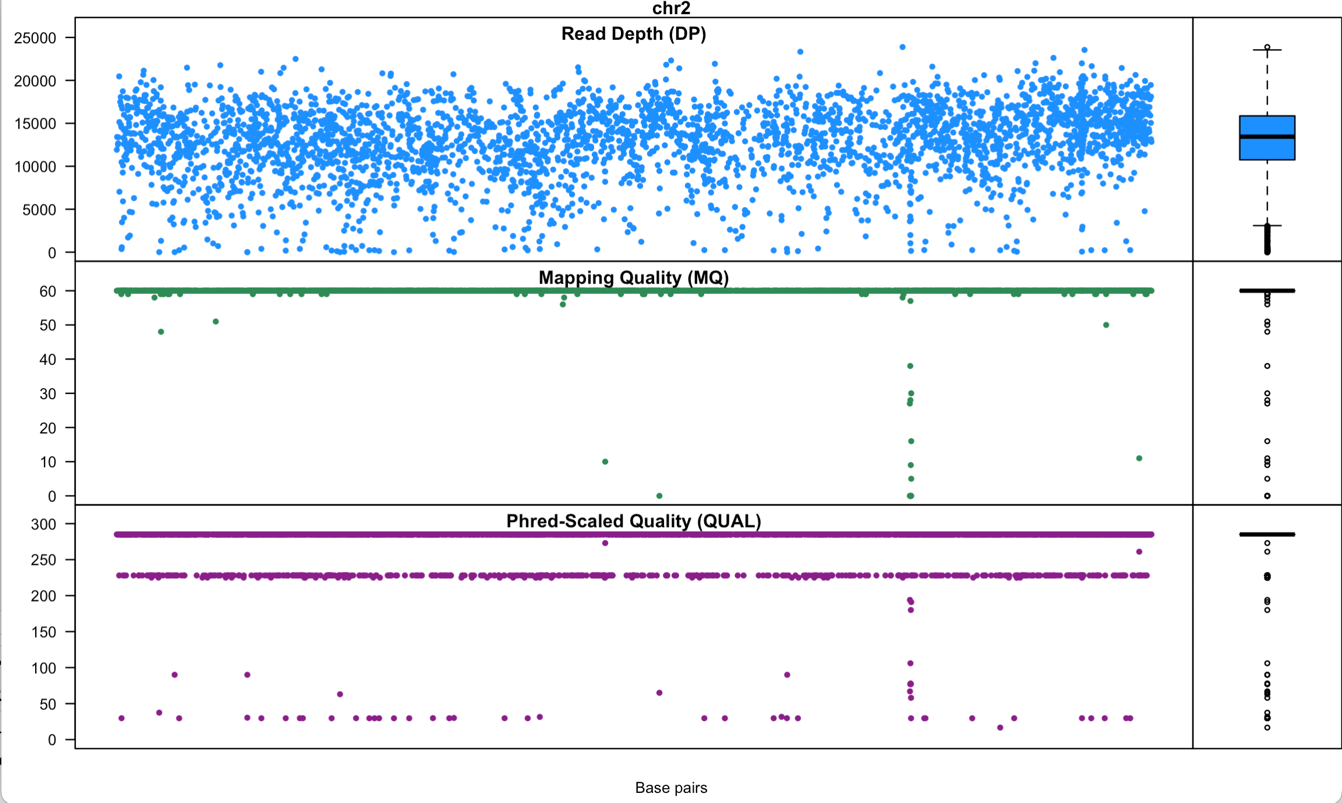


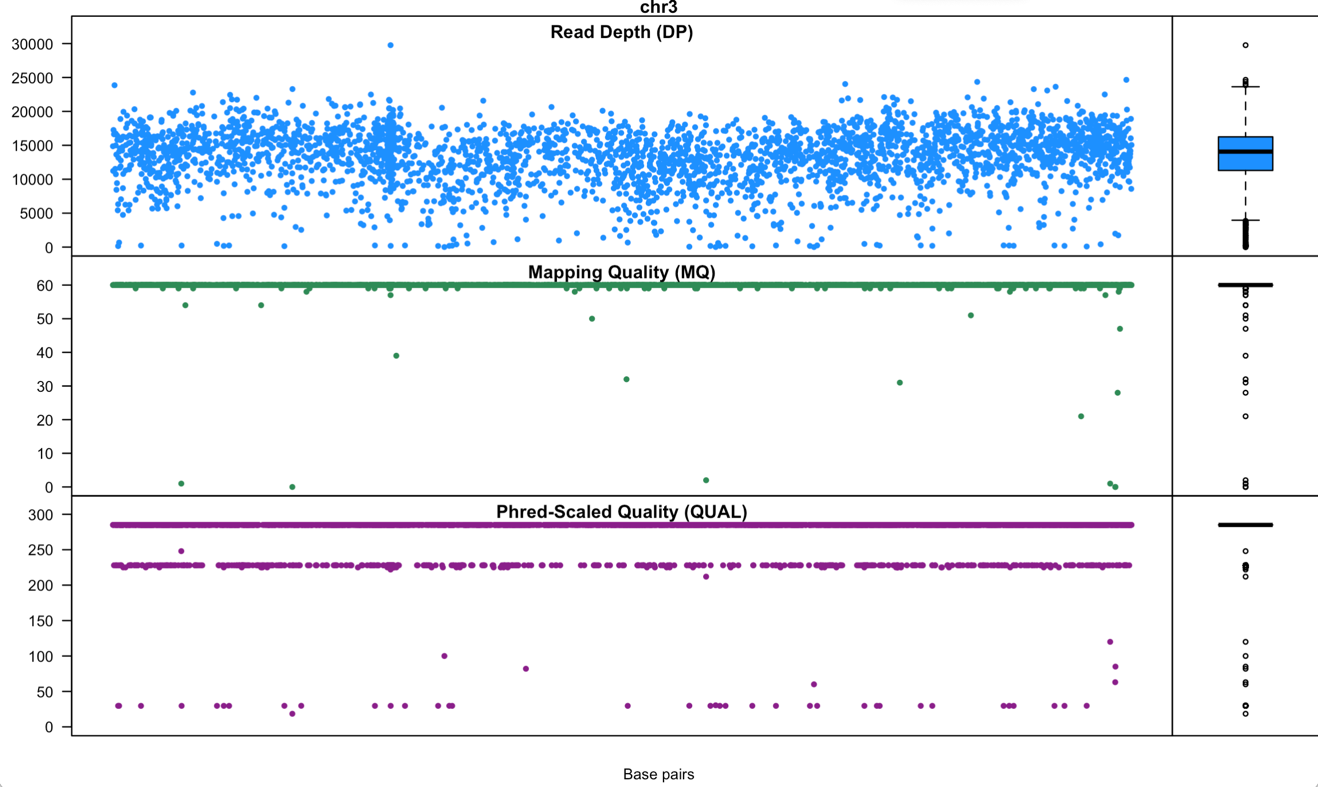


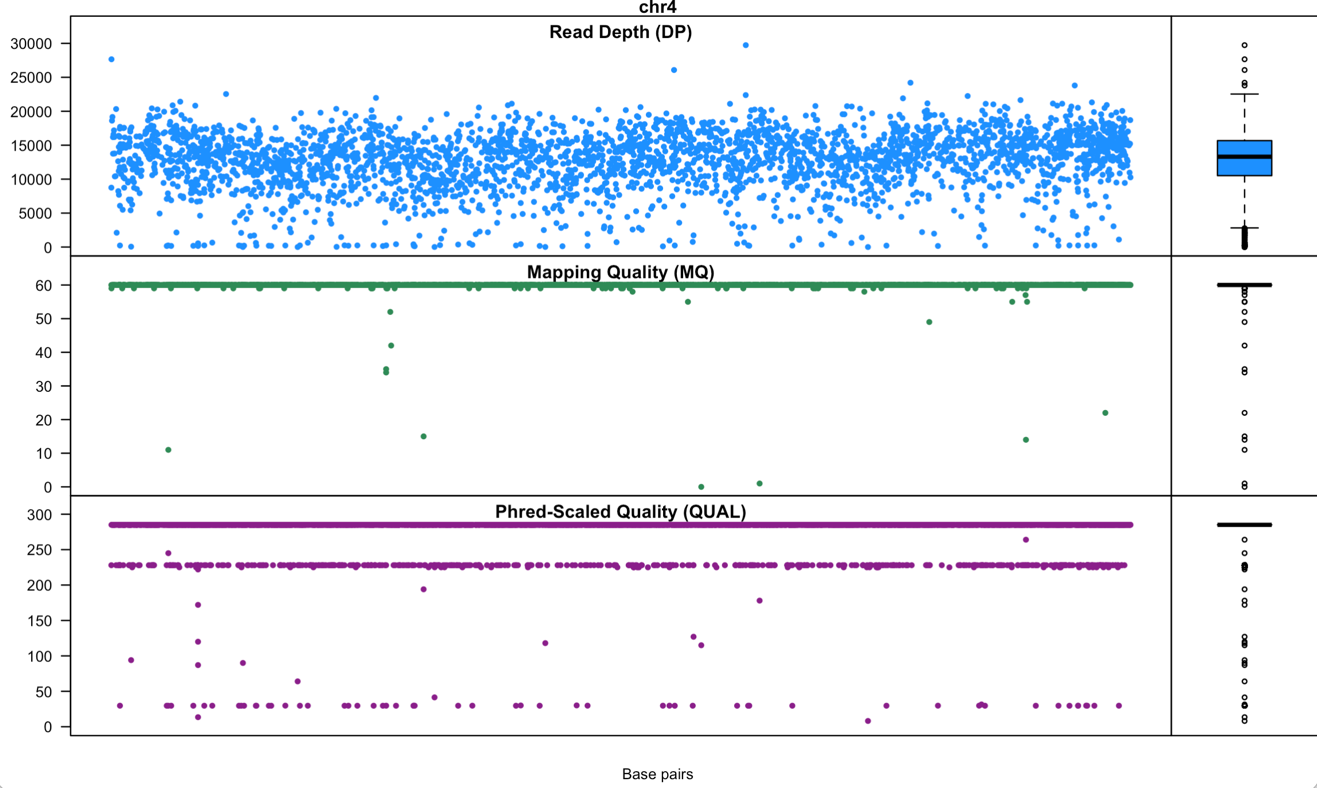


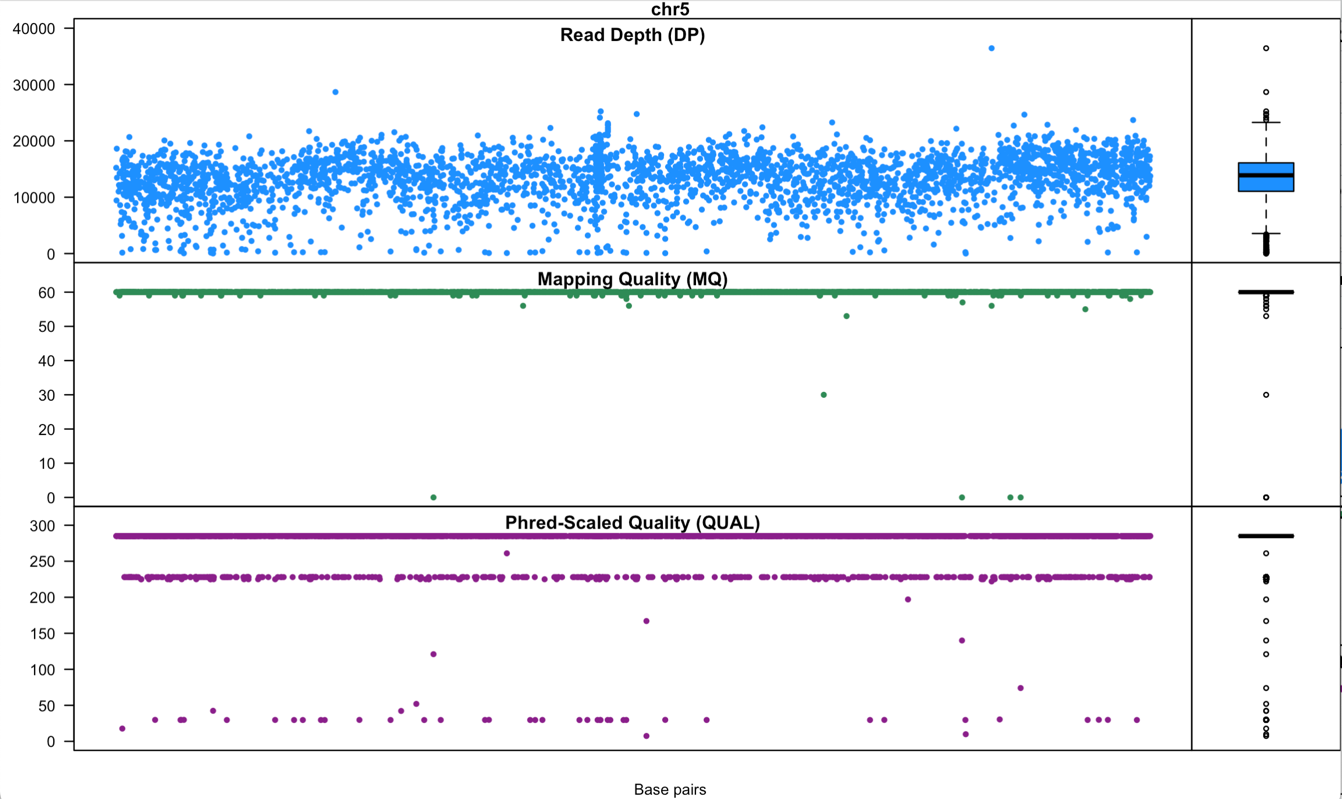


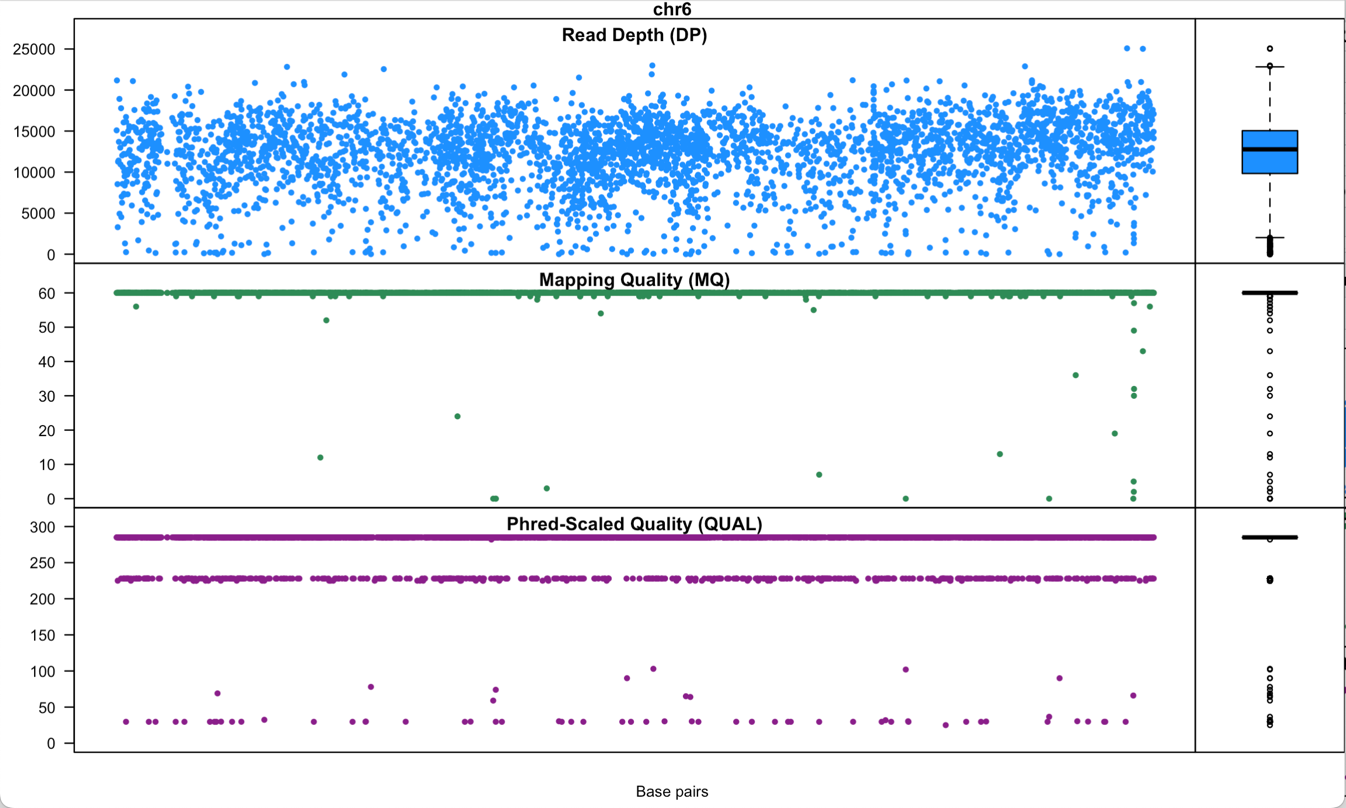


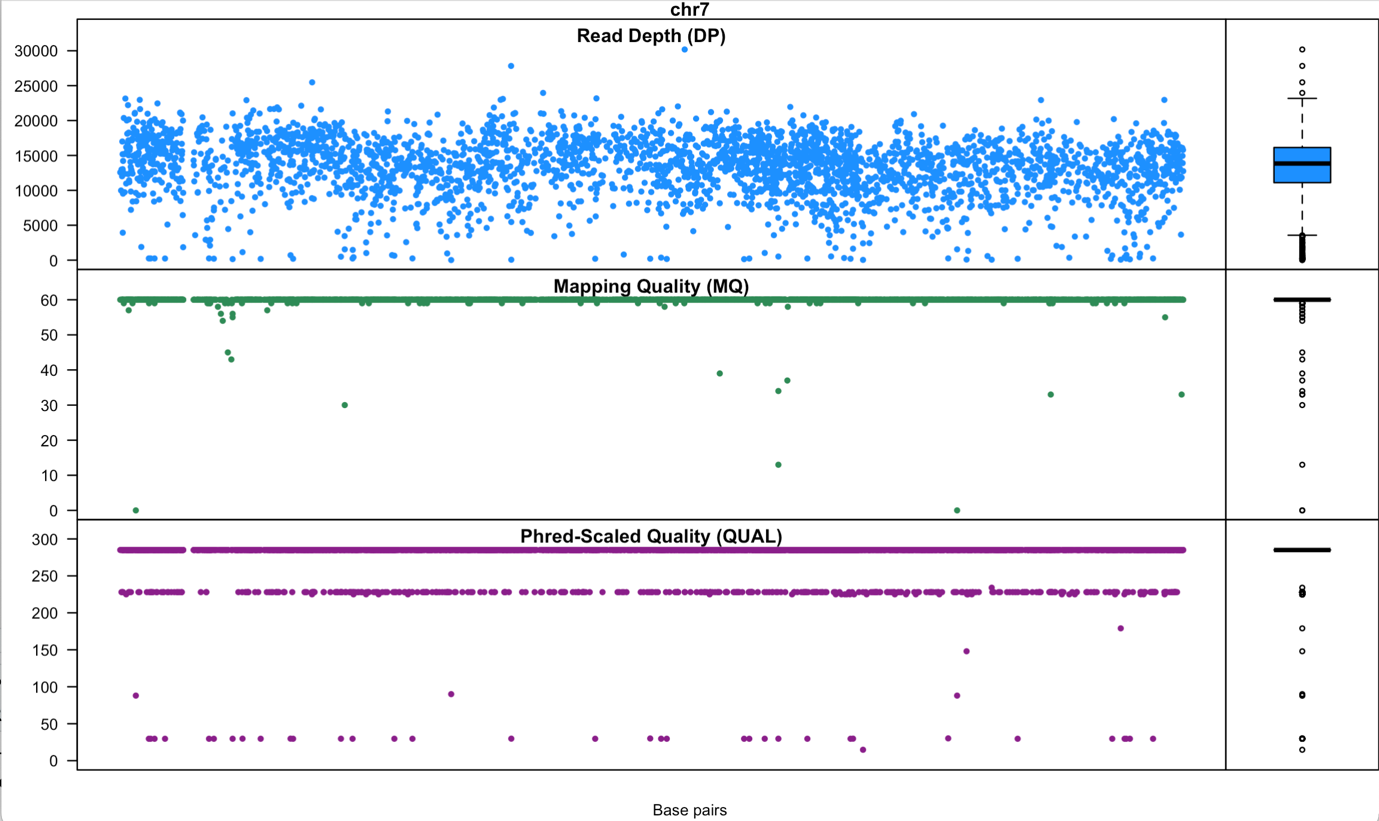


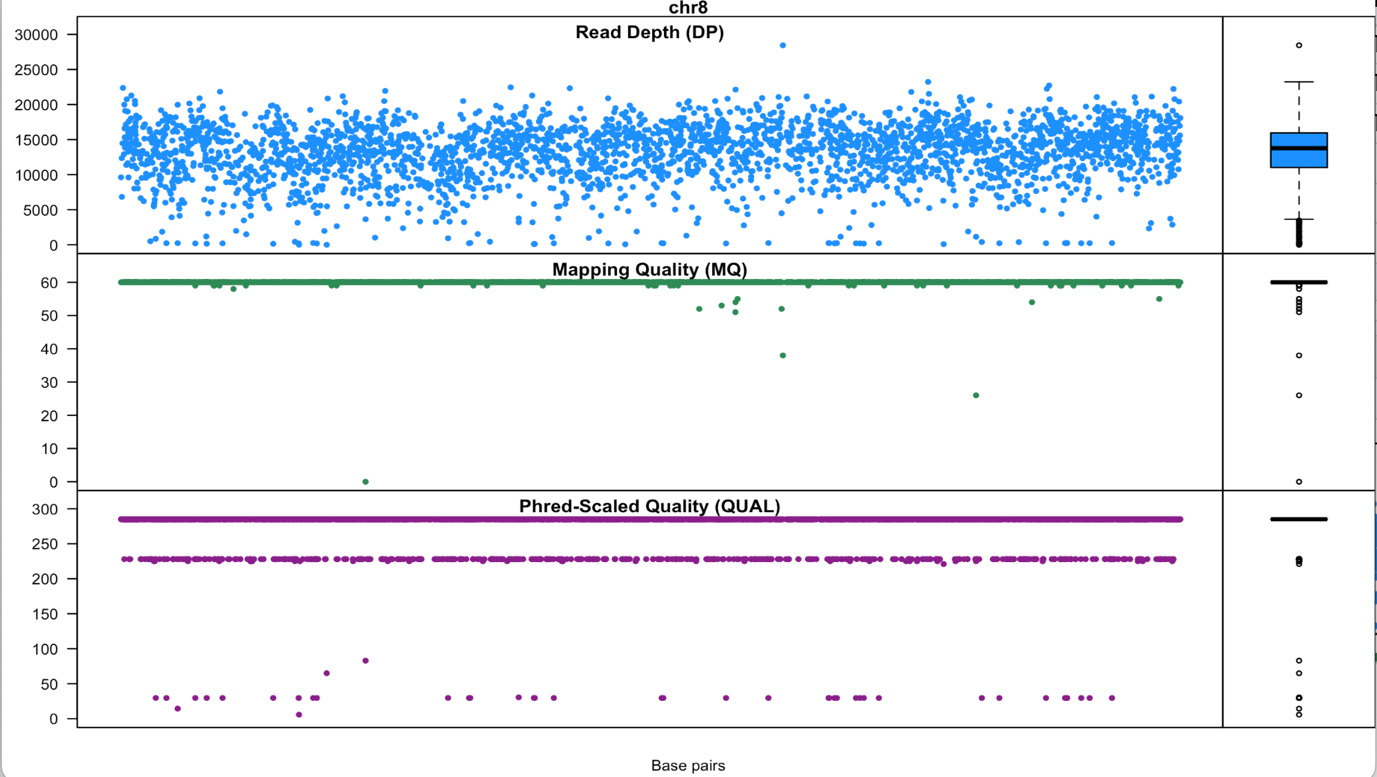


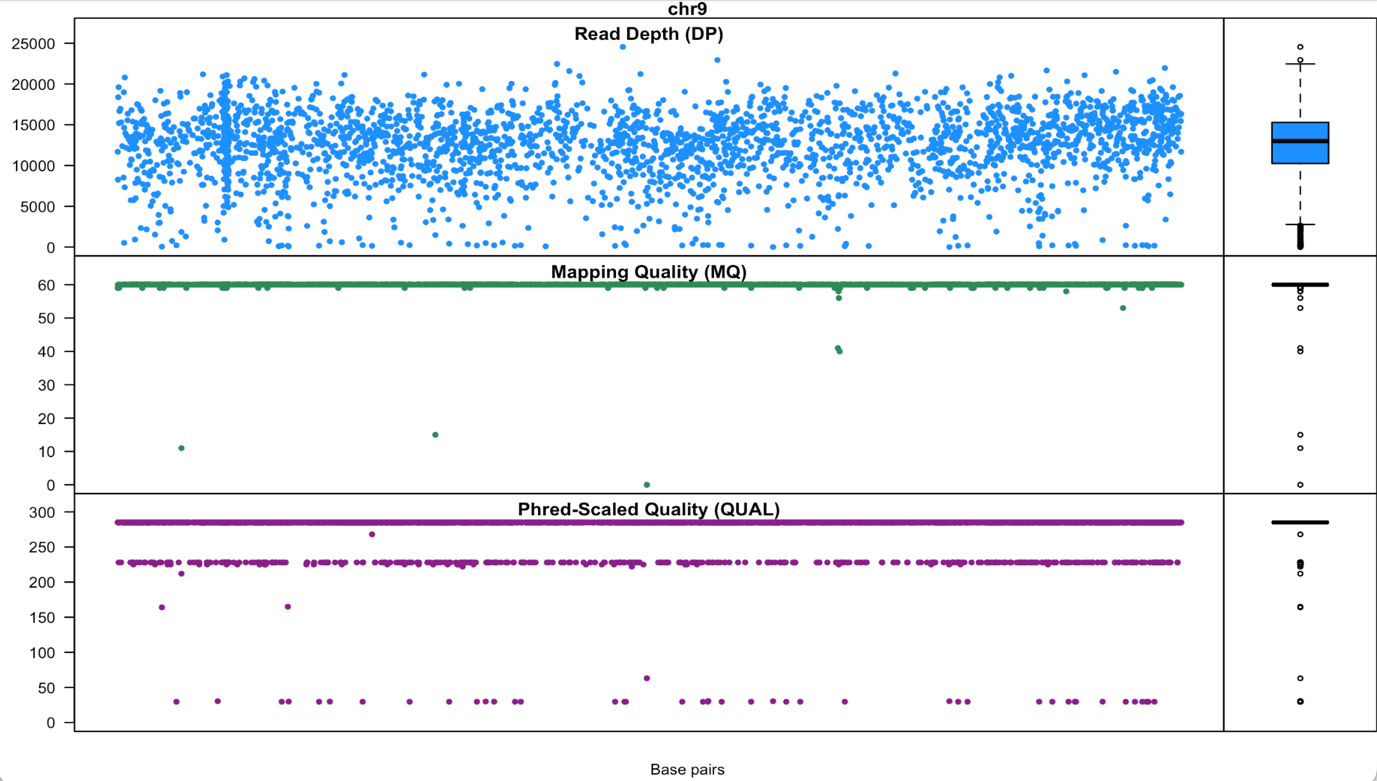


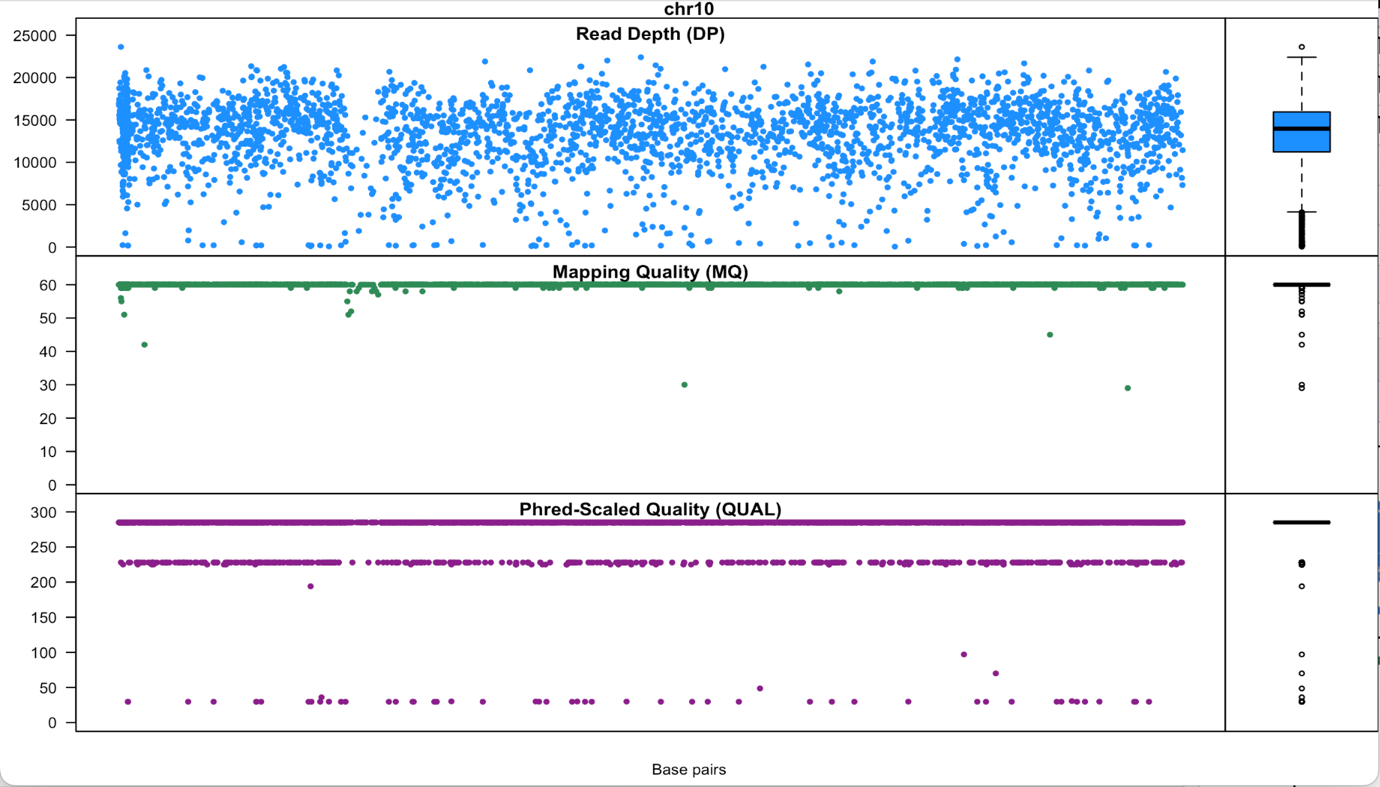


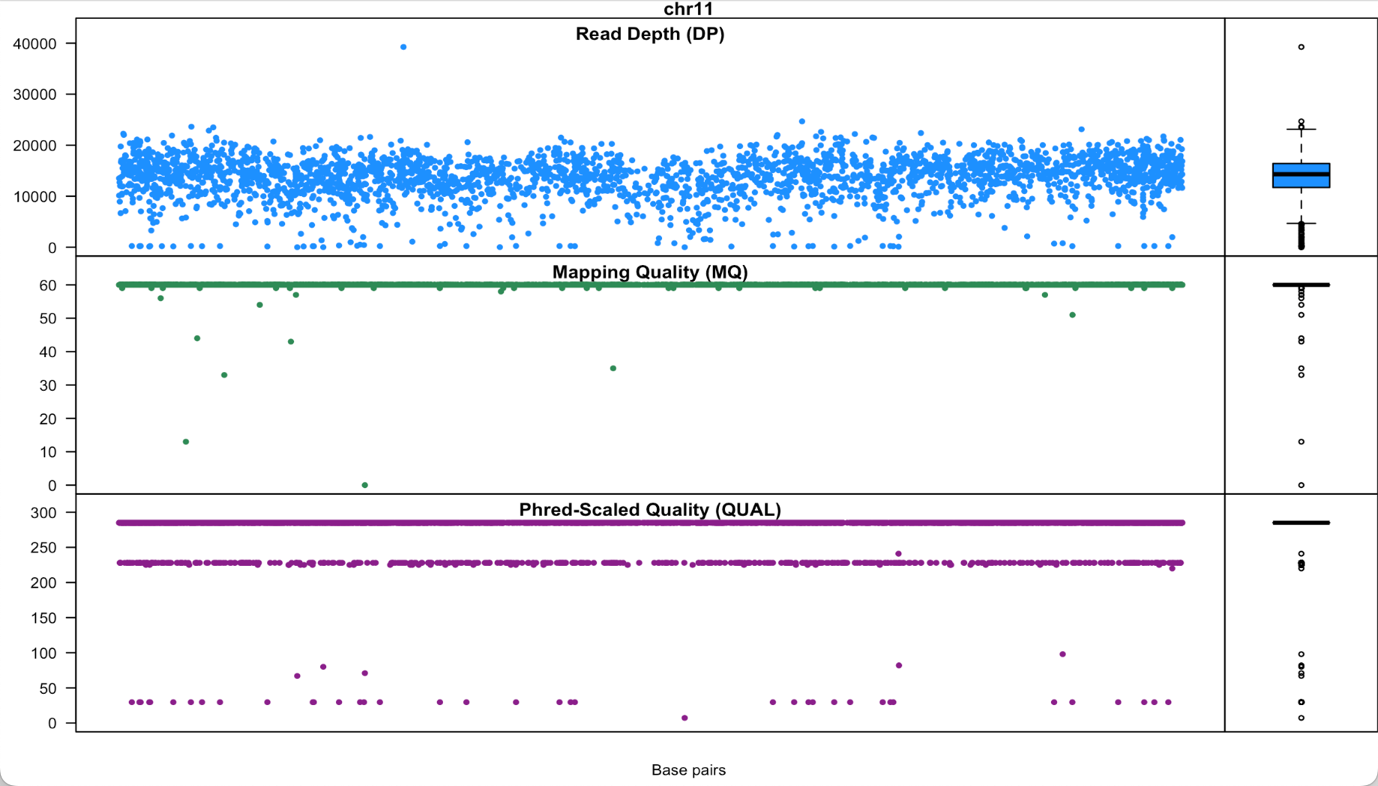


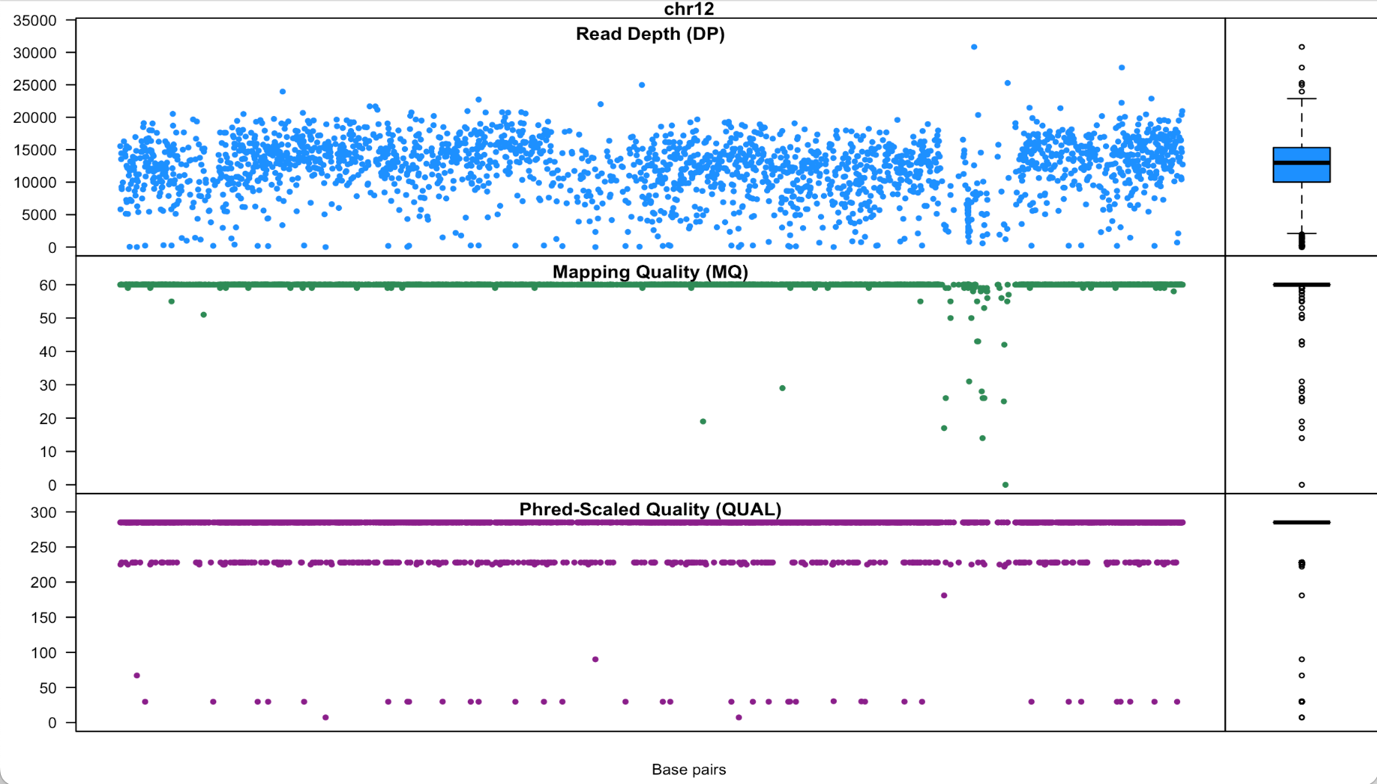


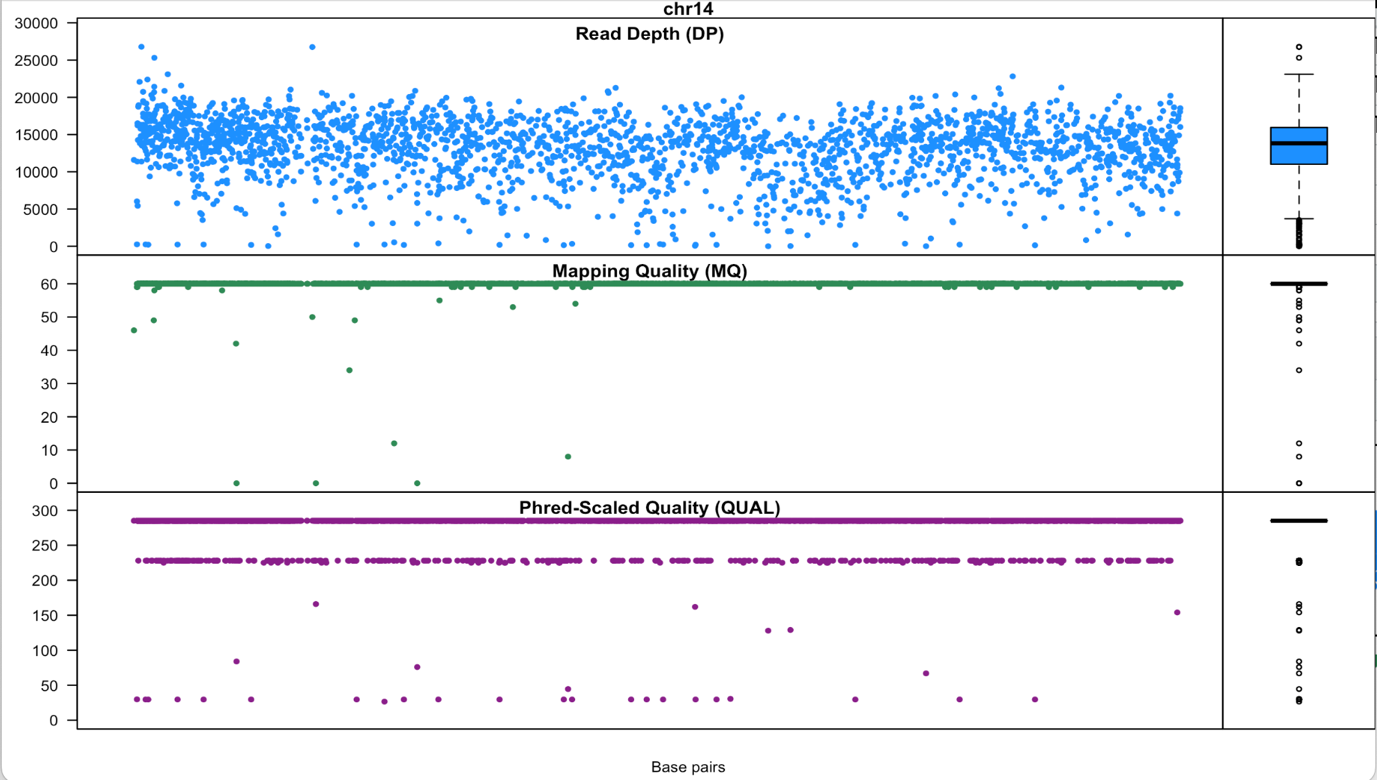


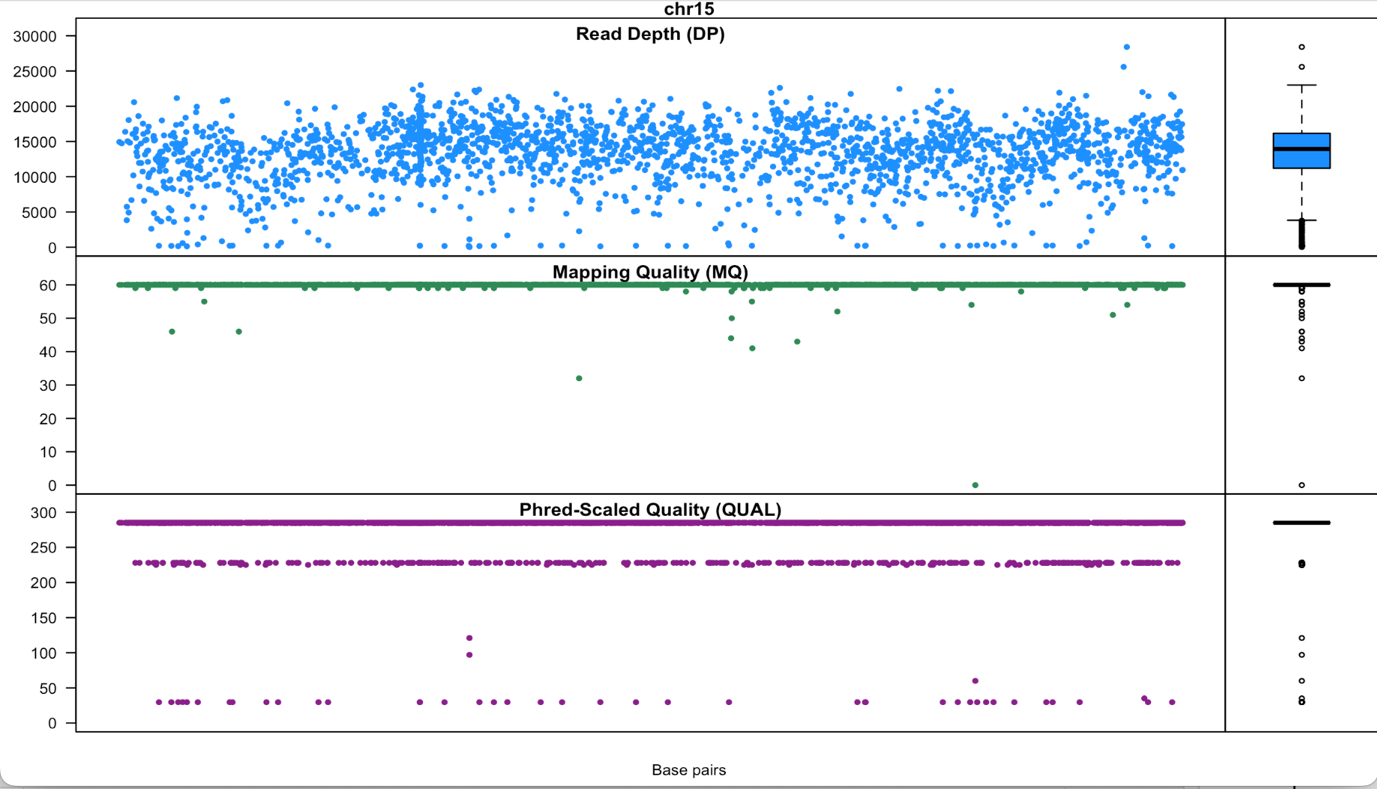


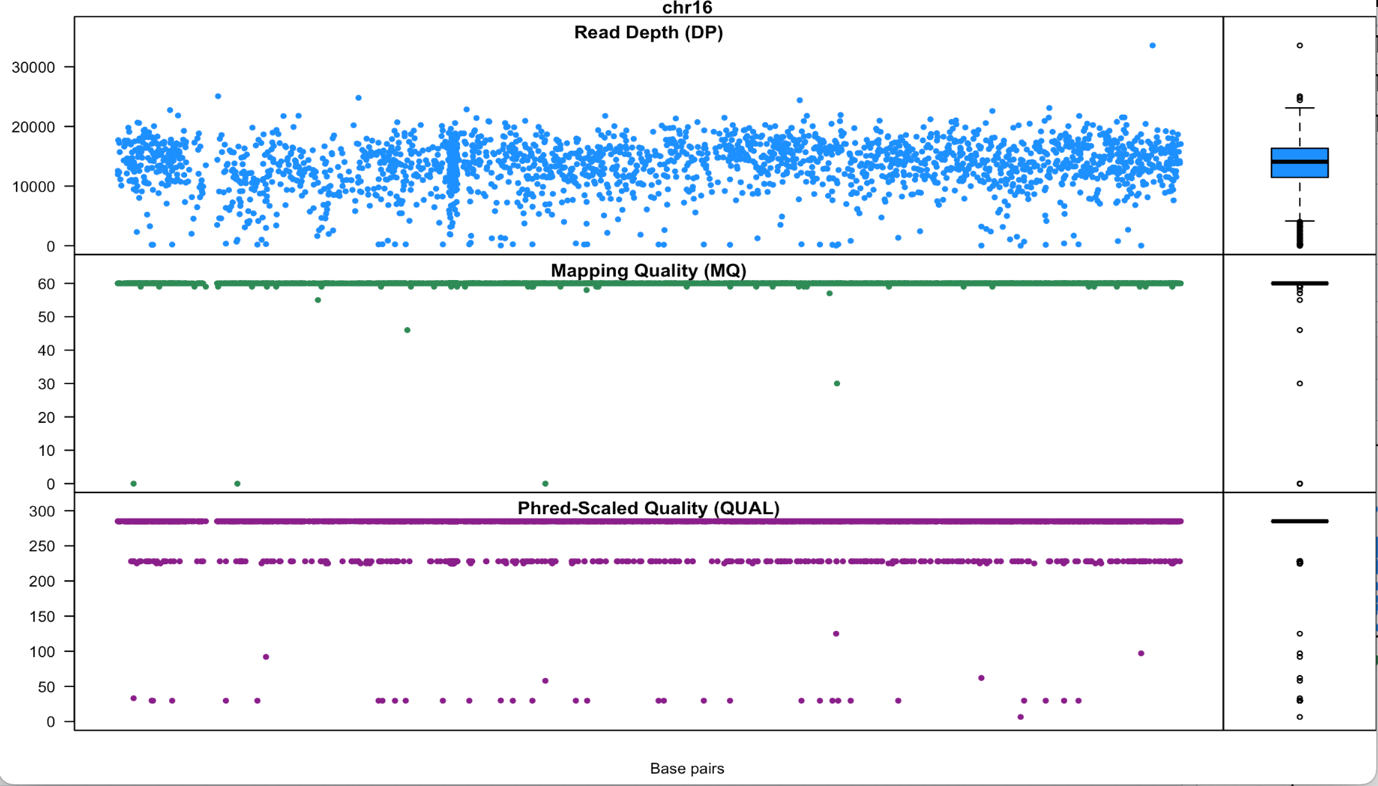


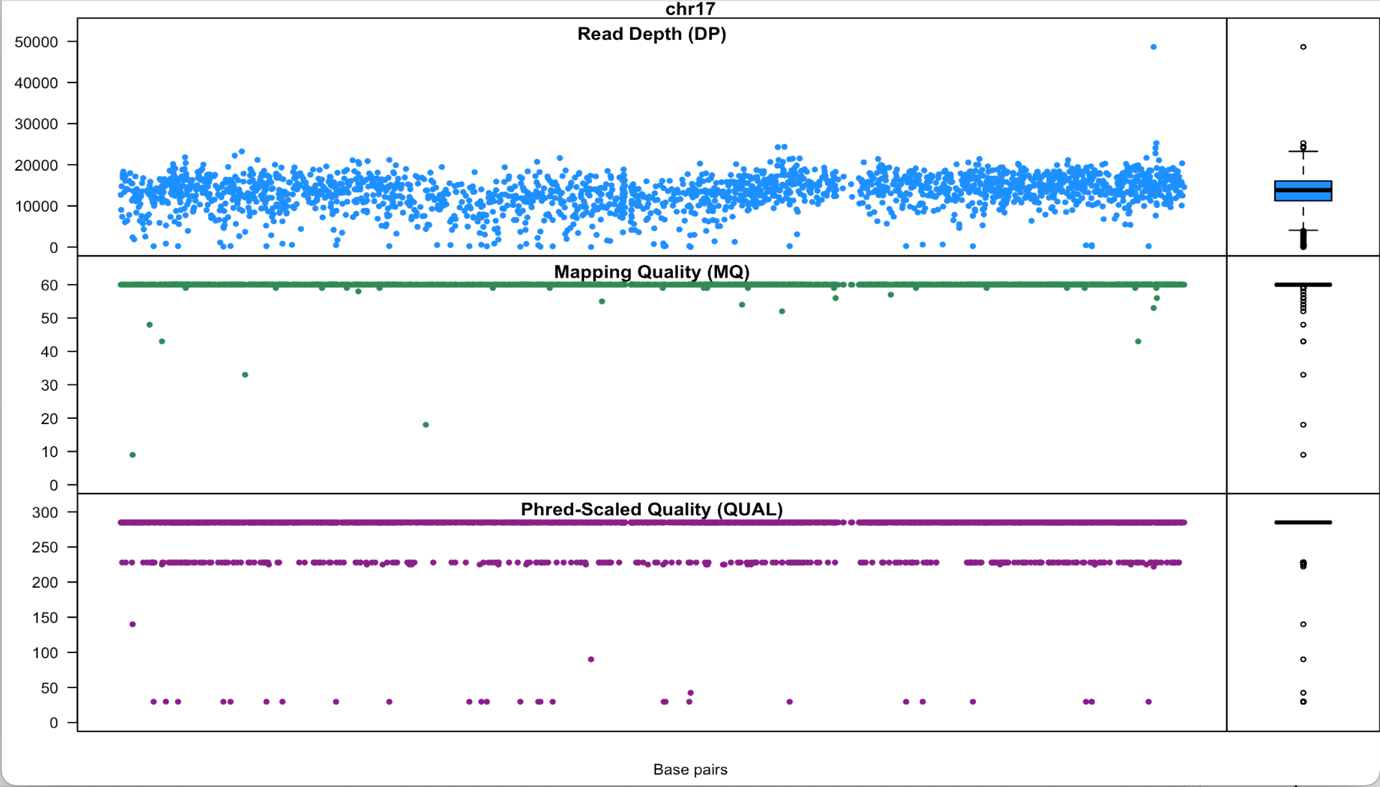


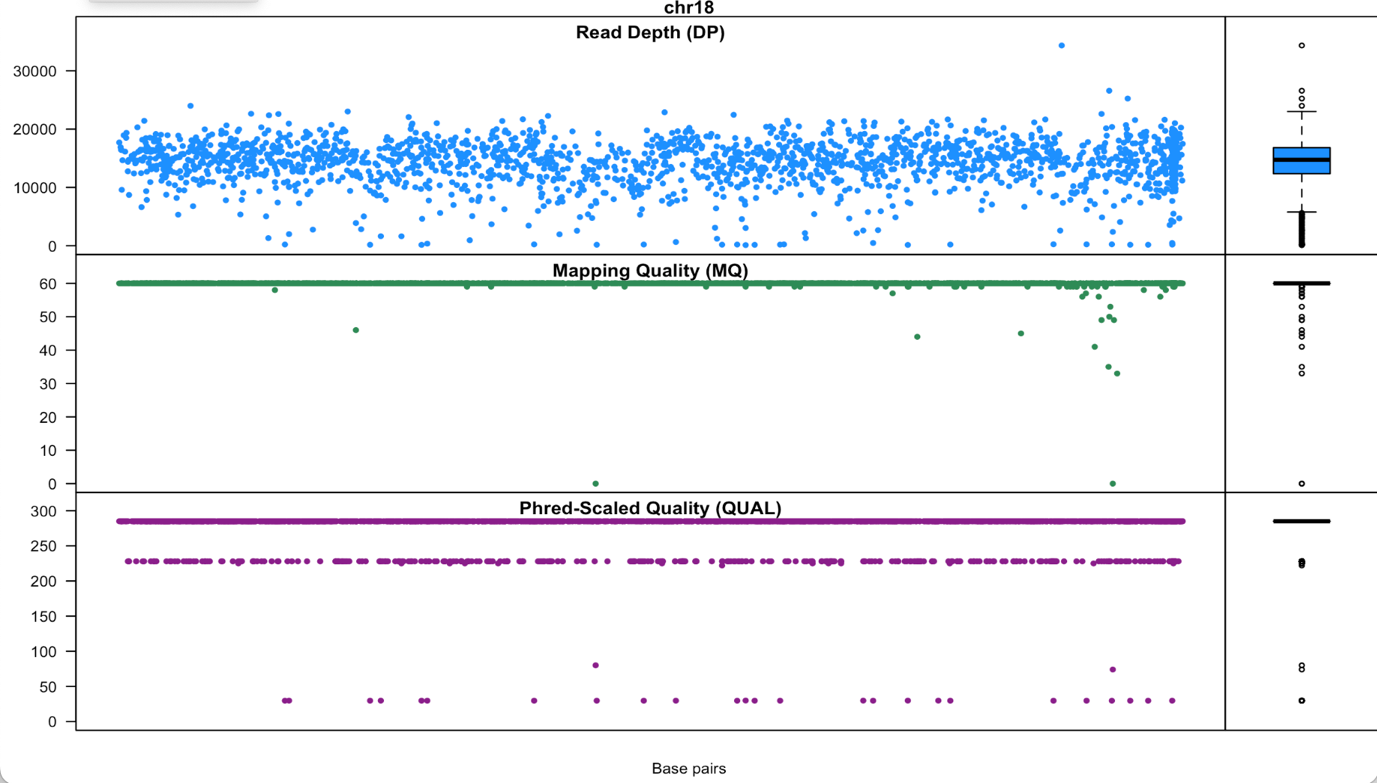


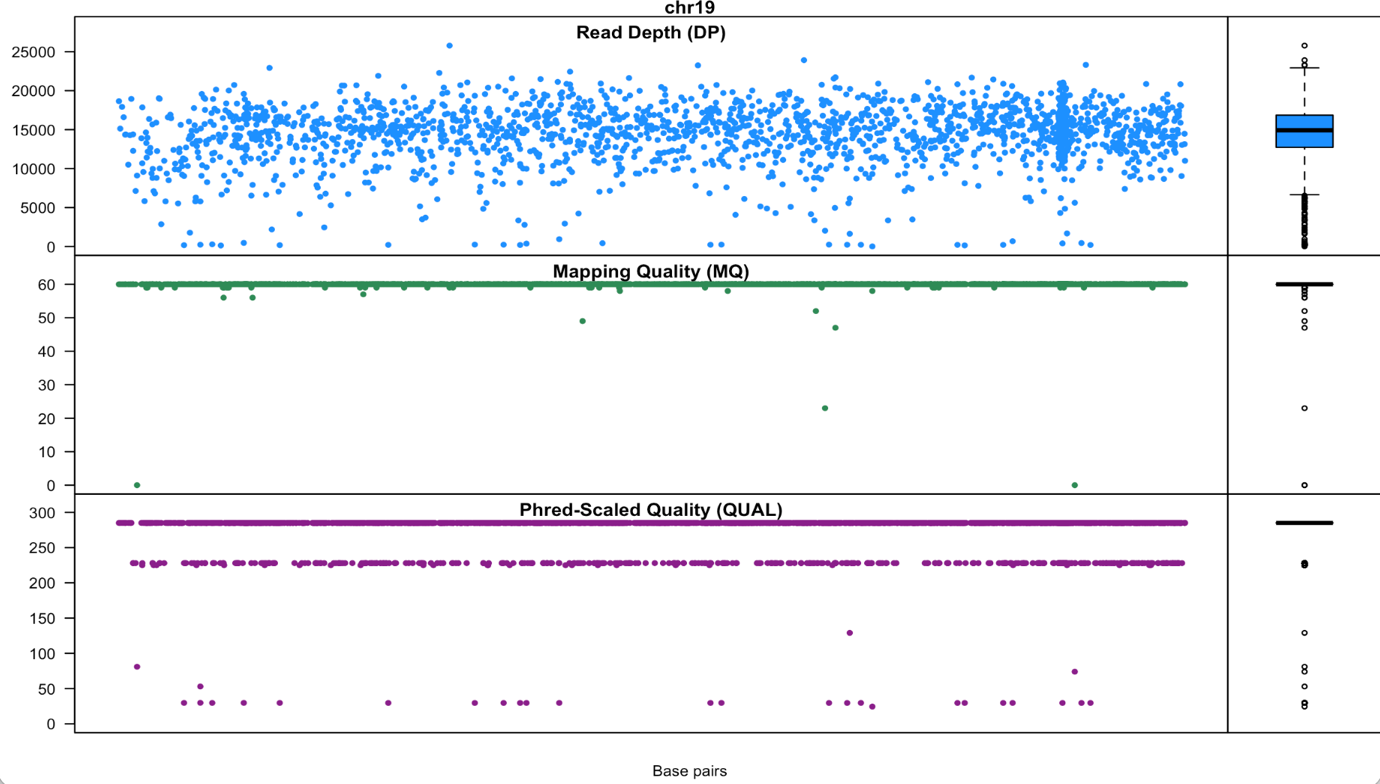


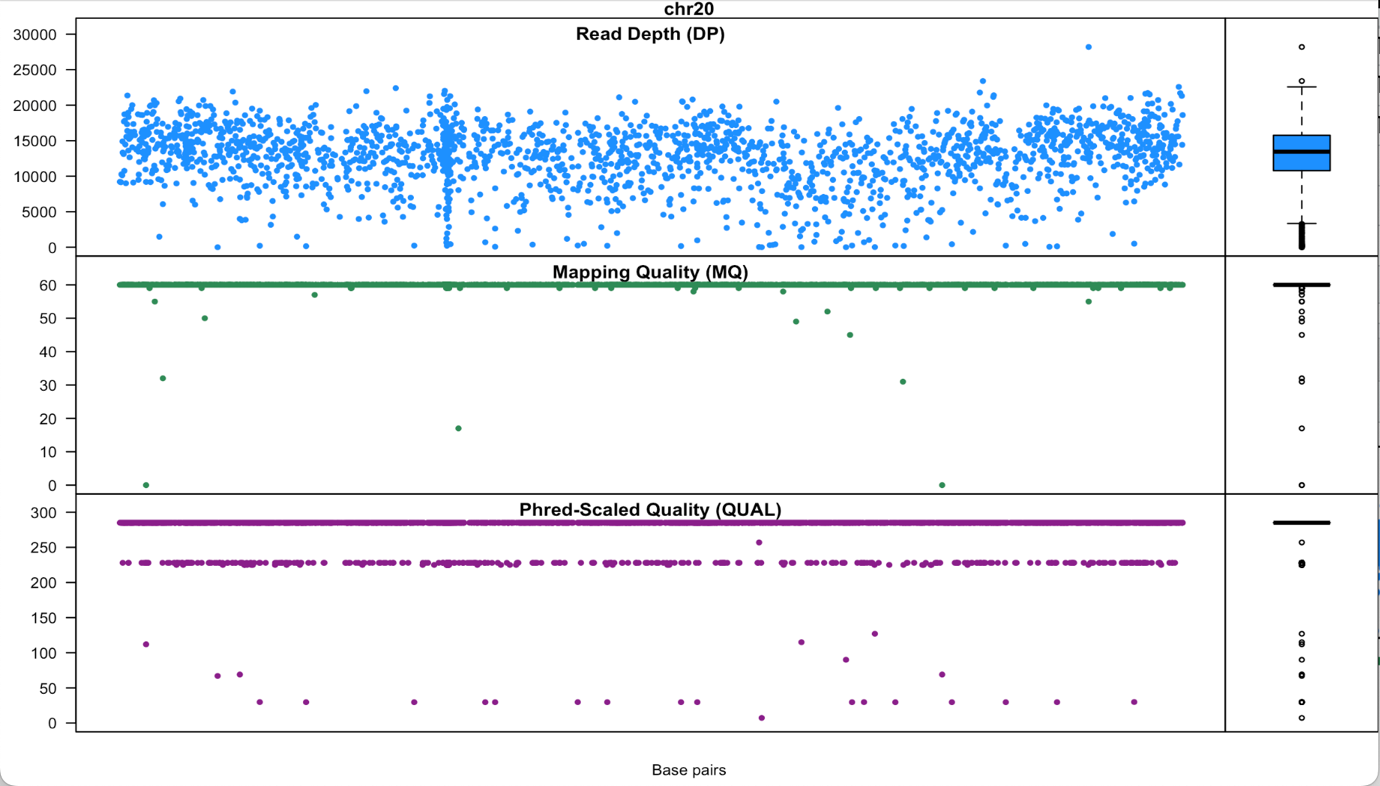


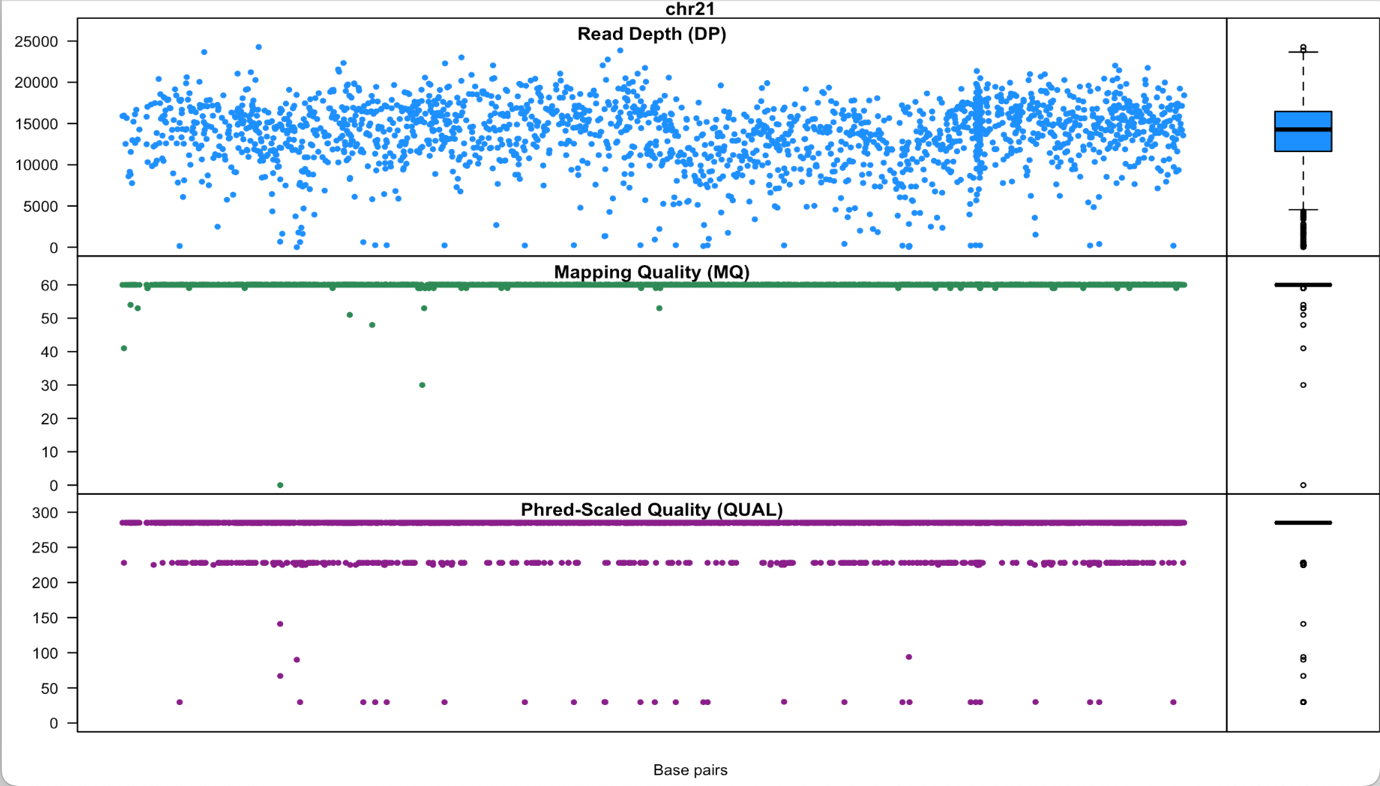


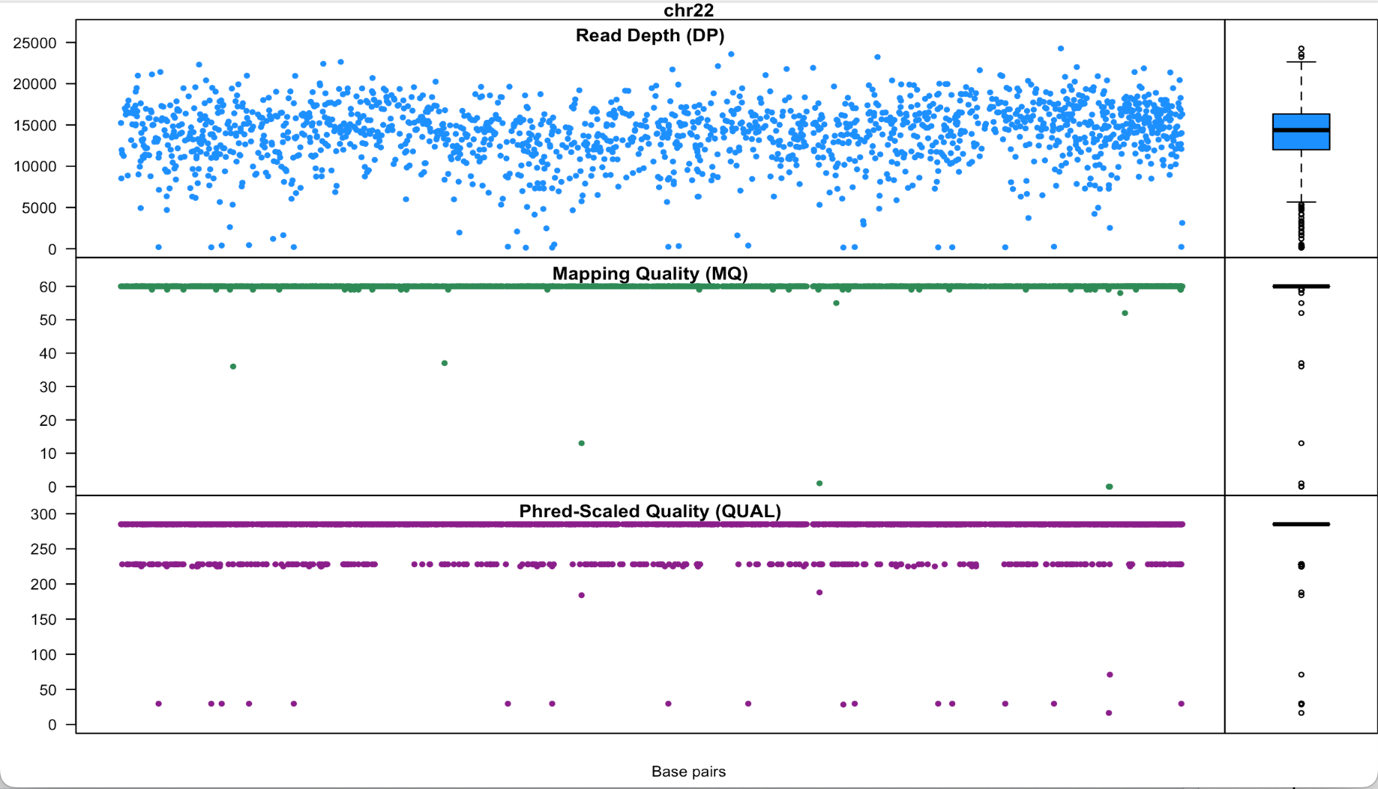


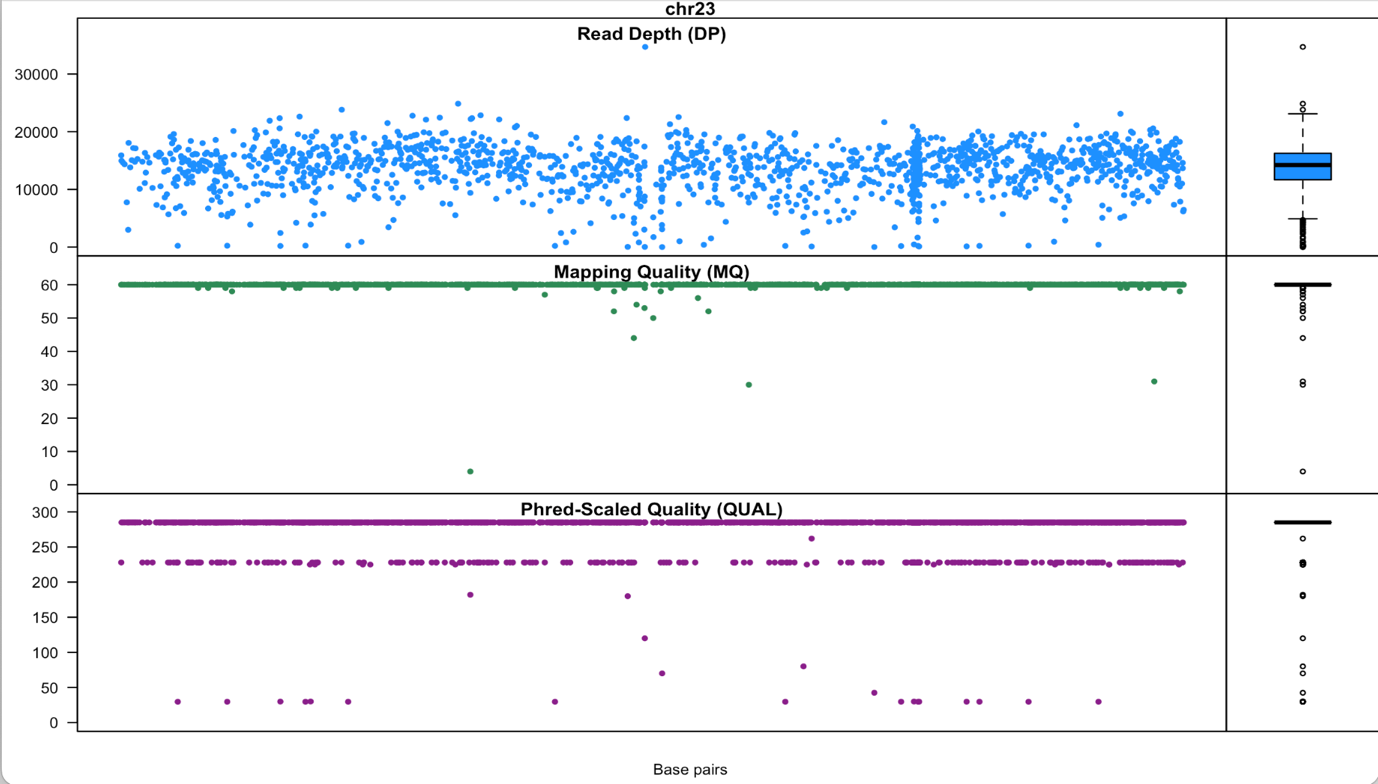


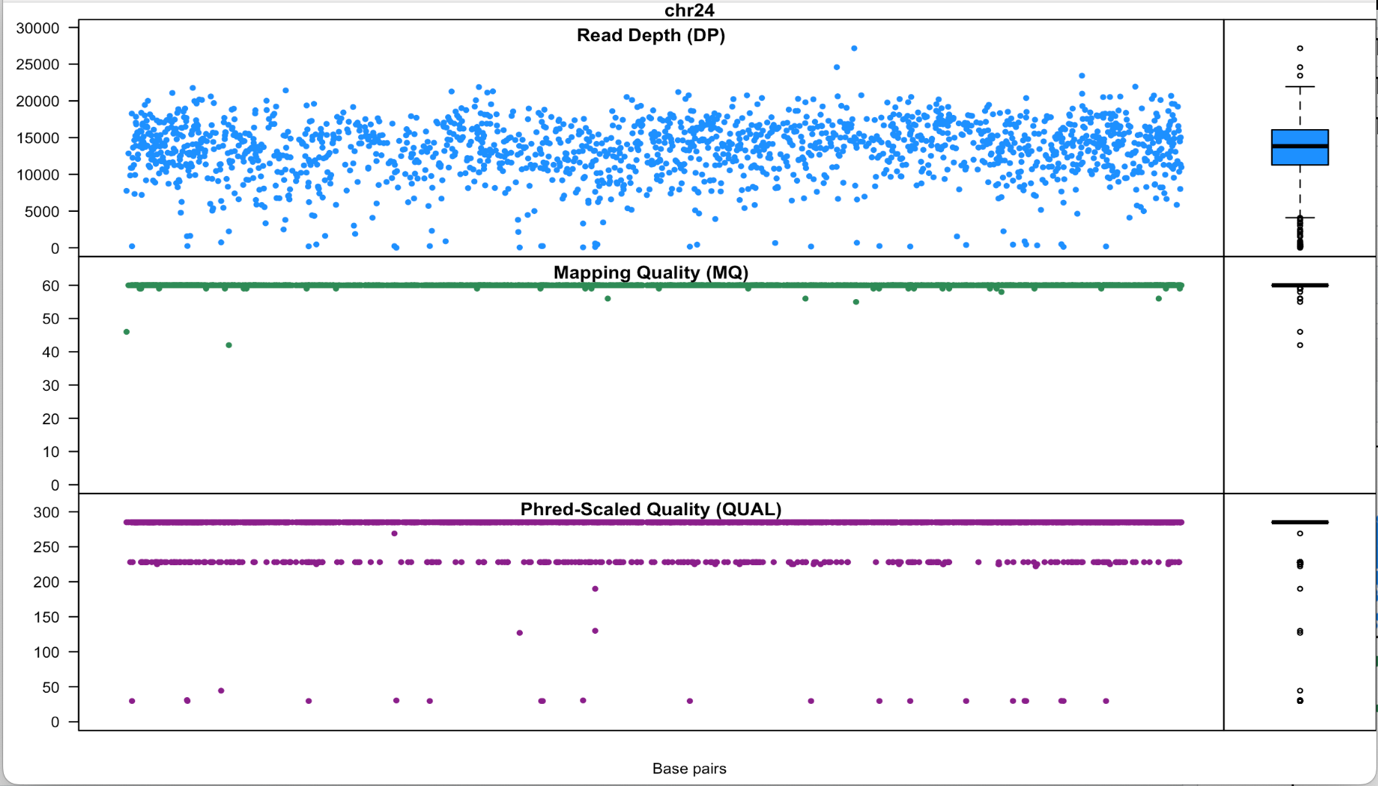


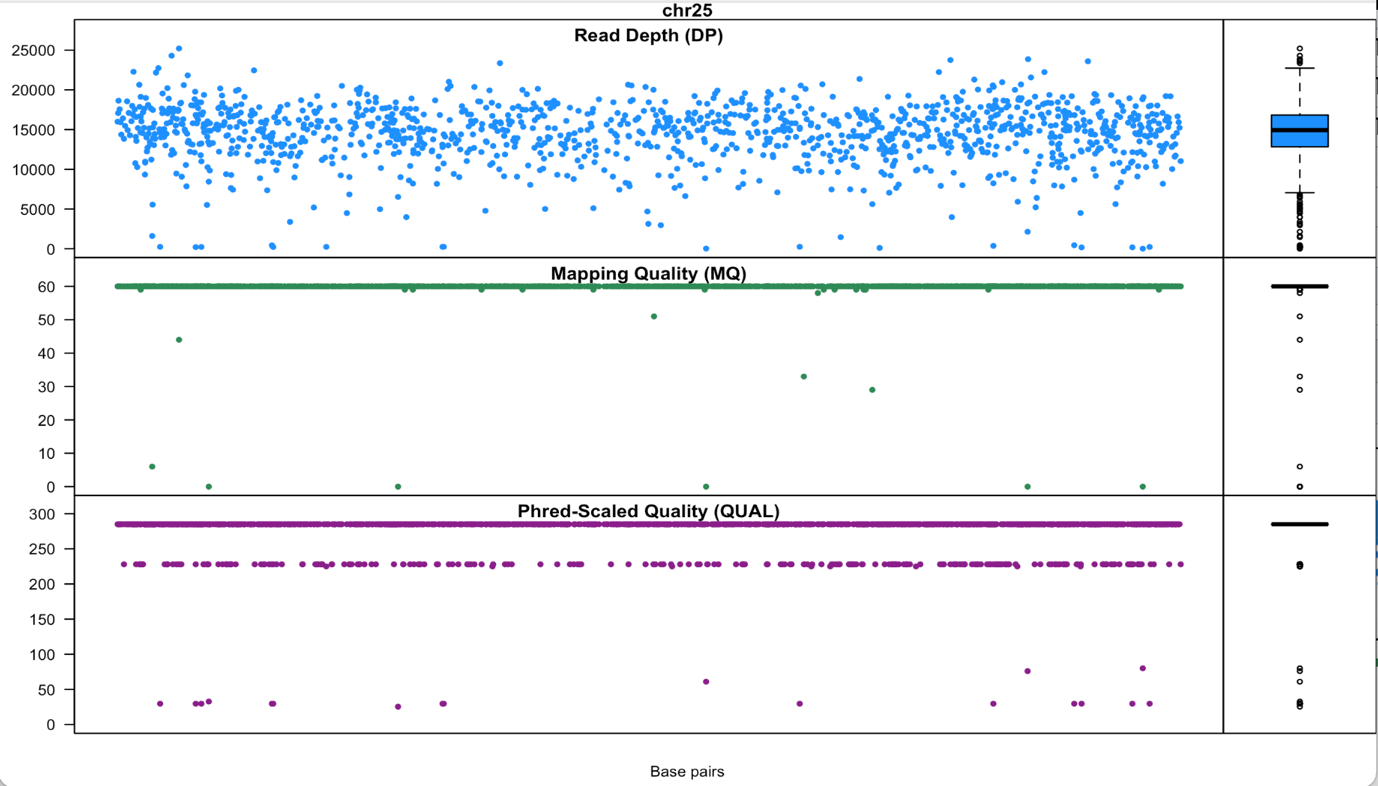


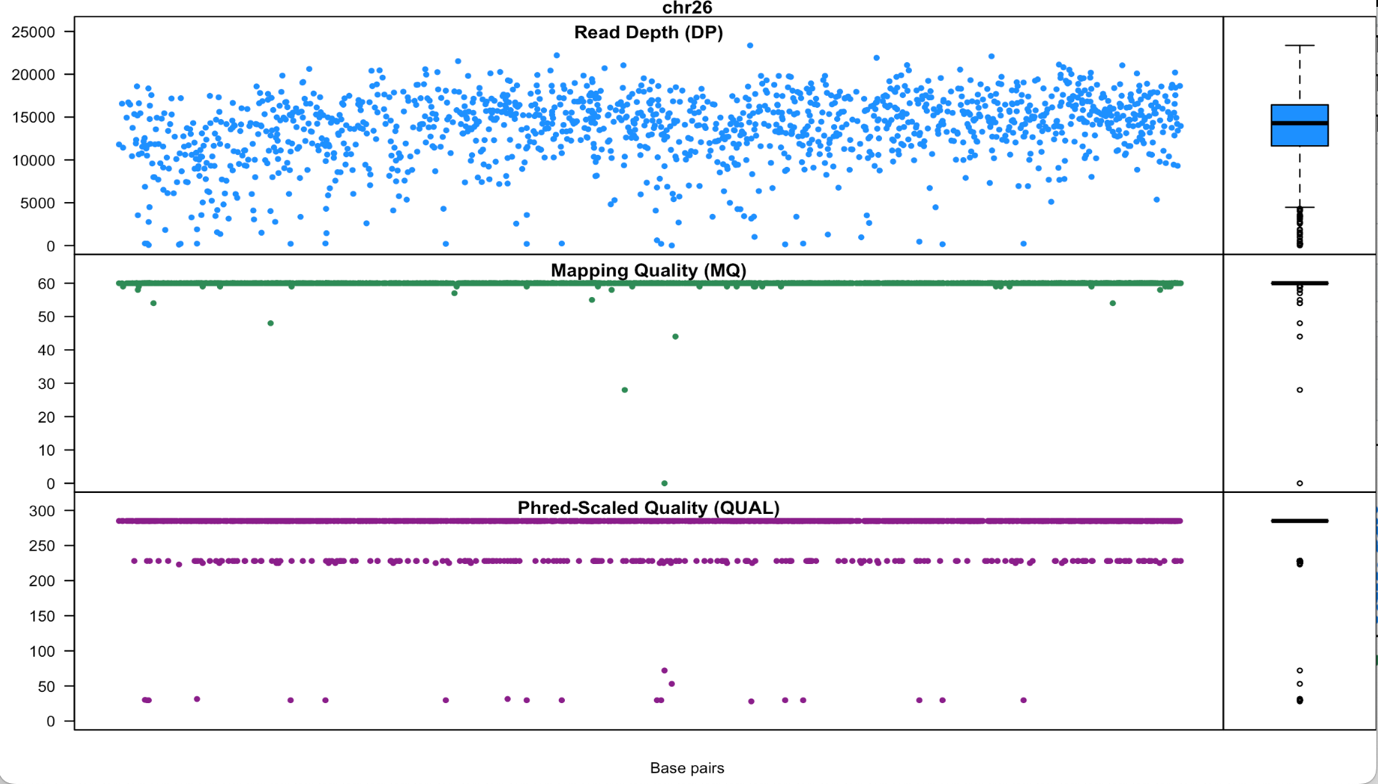


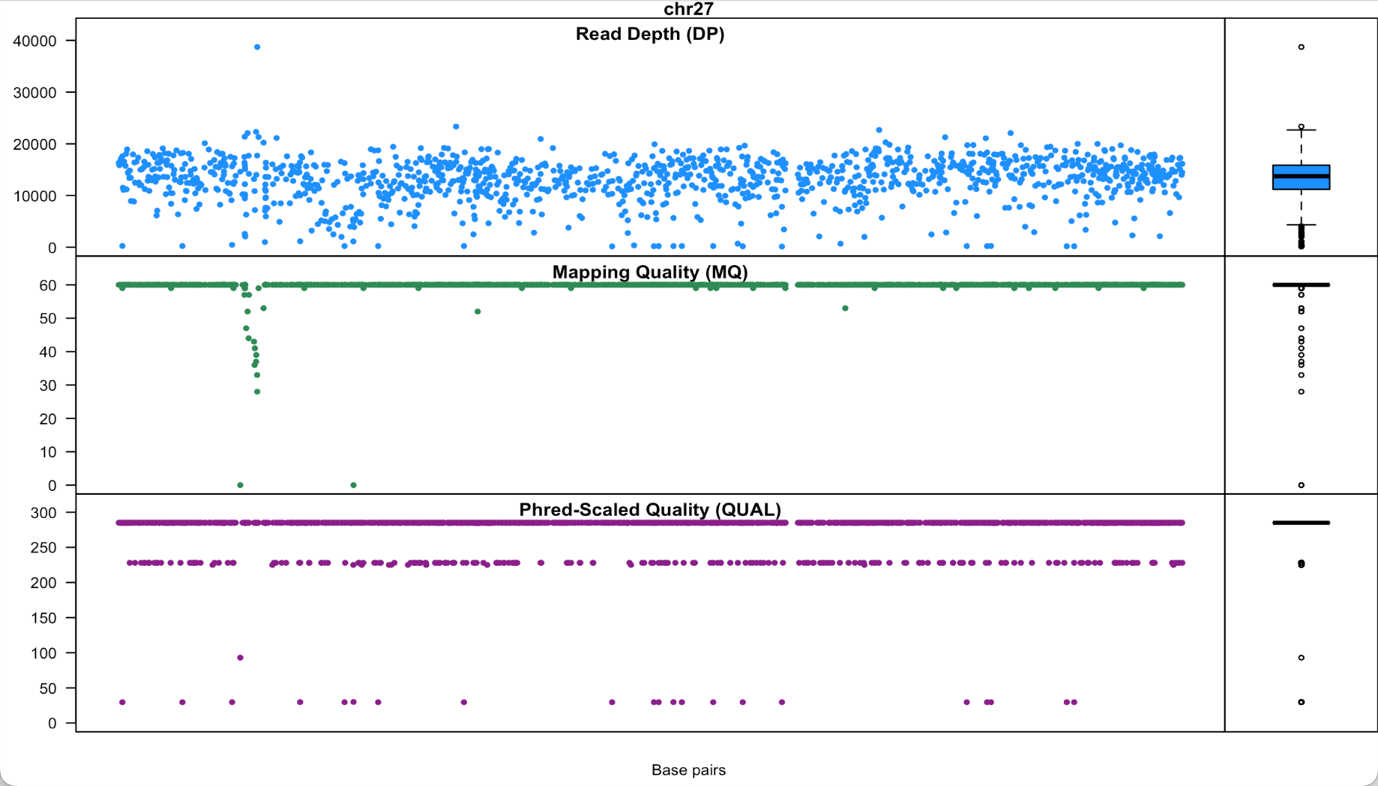


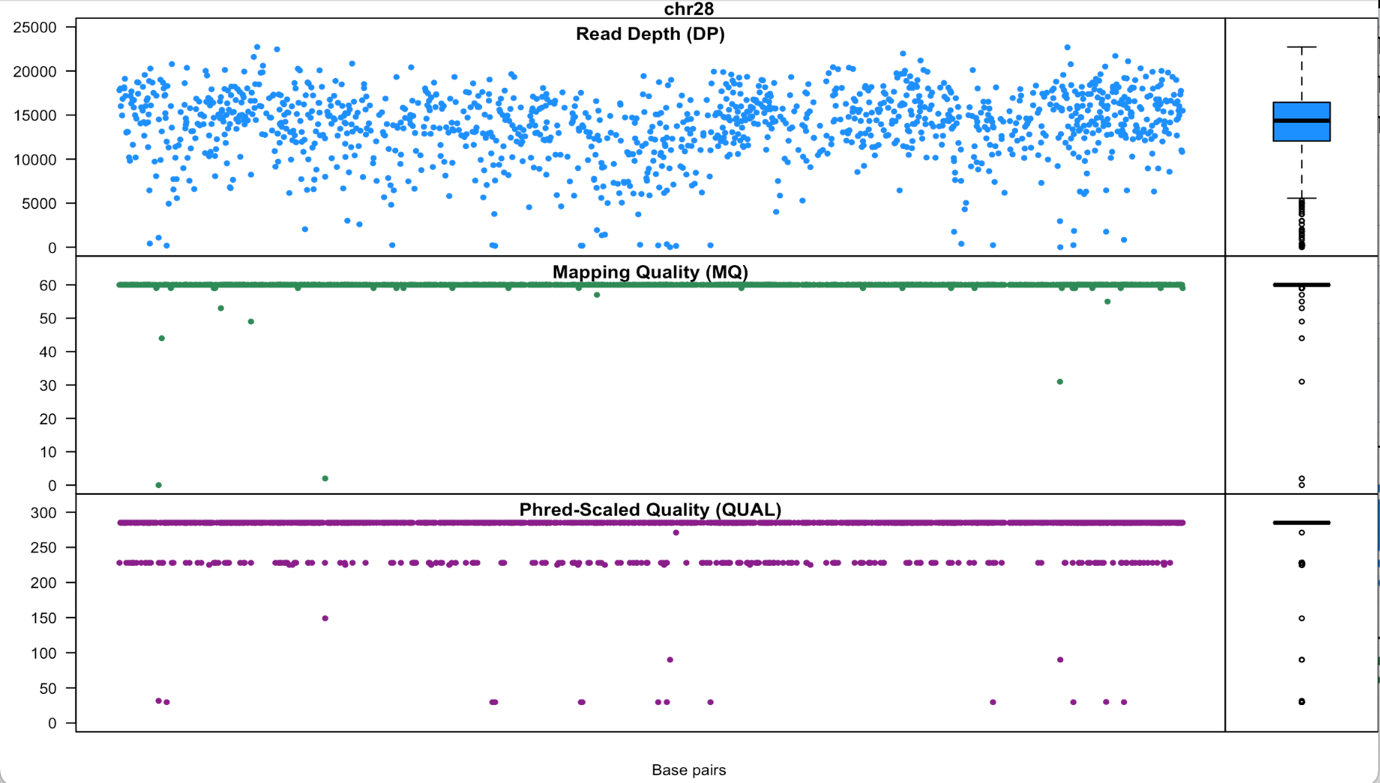


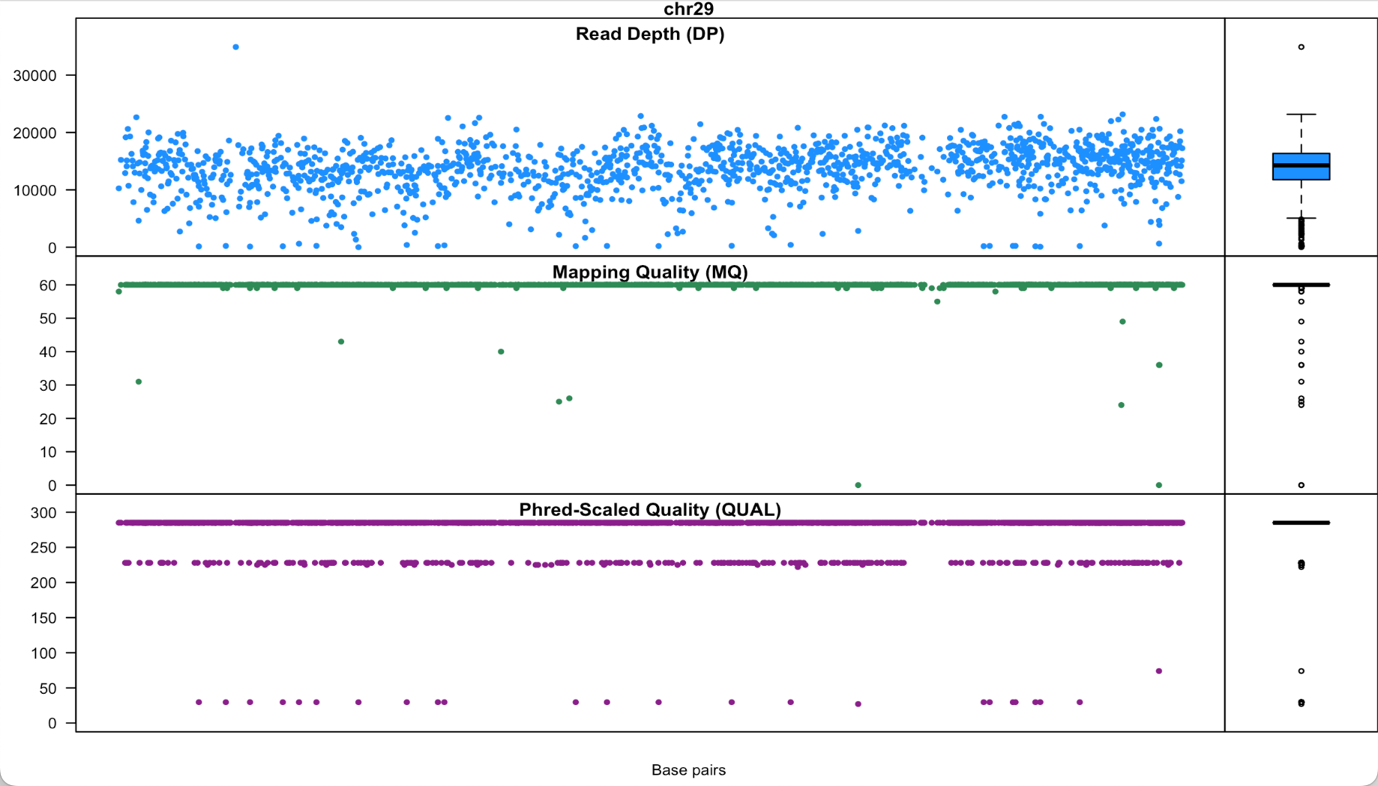


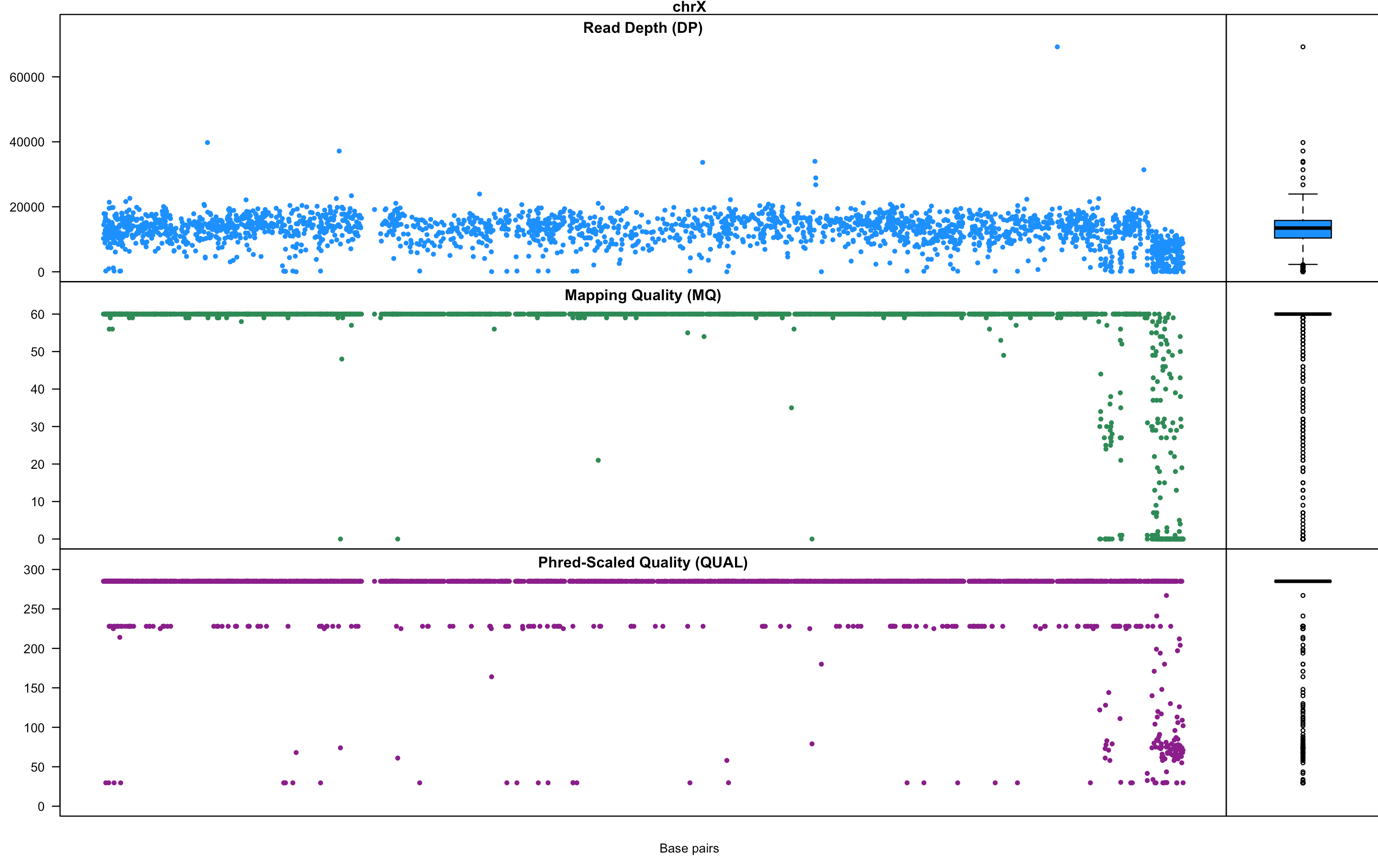


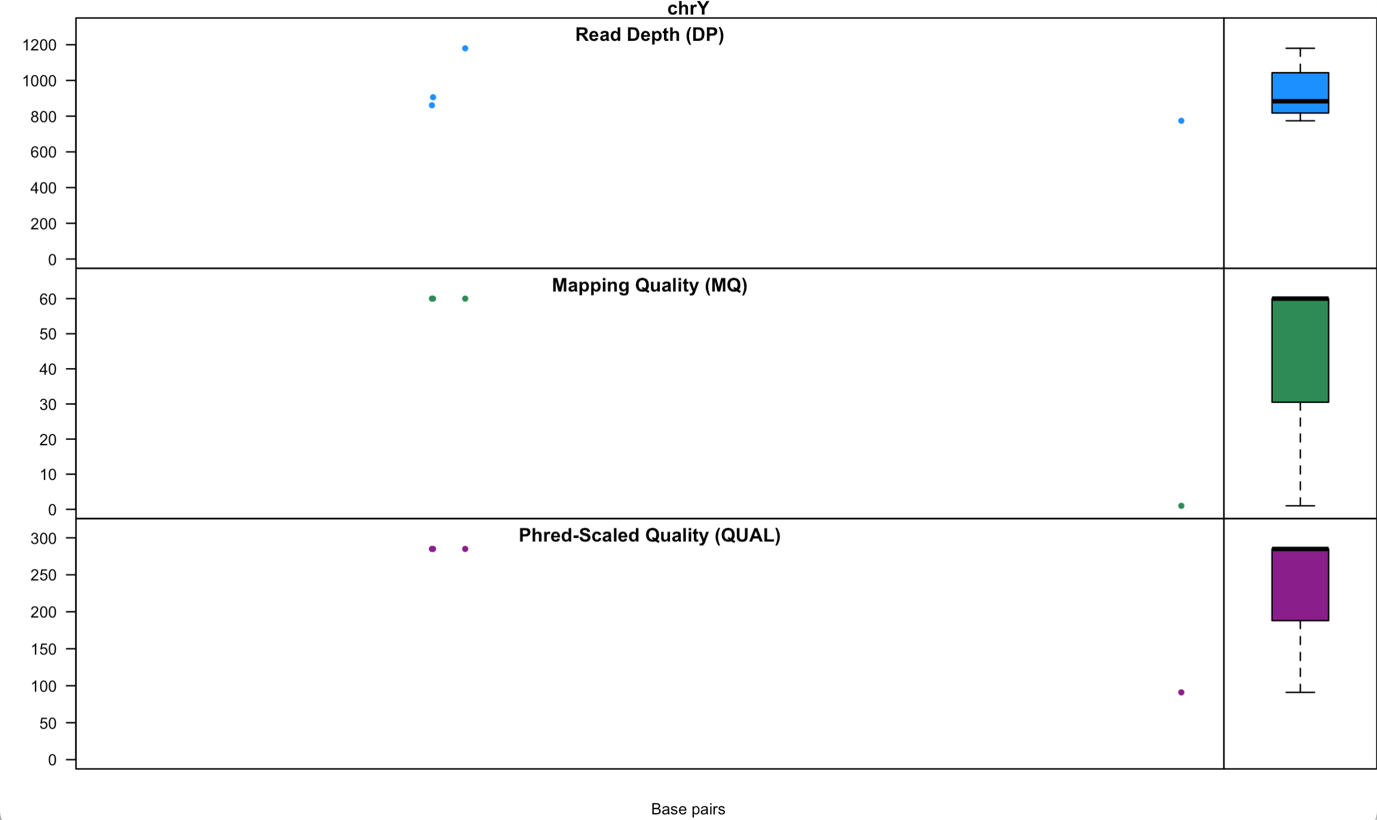


**Figure S7. The distribution of 71553 single nucleotide polymorphisms (SNPs) in TCS, categorized by chromosomal.** This includes 71746 targeted SNPs, with 193 SNPs excluded due to not being called in any sample. The corresponding read depth (DP), mapping quality (MQ), and phred-scaled quality (QUAL) are displayed.
