# Supplementary material for: Evaluating the Efficacy of Target Capture Sequencing for Genotyping in Cattle
Source: Genes (Basel). 2024 Sep 18;15(9):1218. doi: 10.3390/genes15091218 (PMC11431841; doi:10.3390/genes15091218)
Supplement: Supplementary file 1 [file genes-15-01218-s001.zip › Probe_capture_paper_supplementary_files_20240910/Sub_Figures/FigureS4_call rates of TCS and Versa50K .docx]

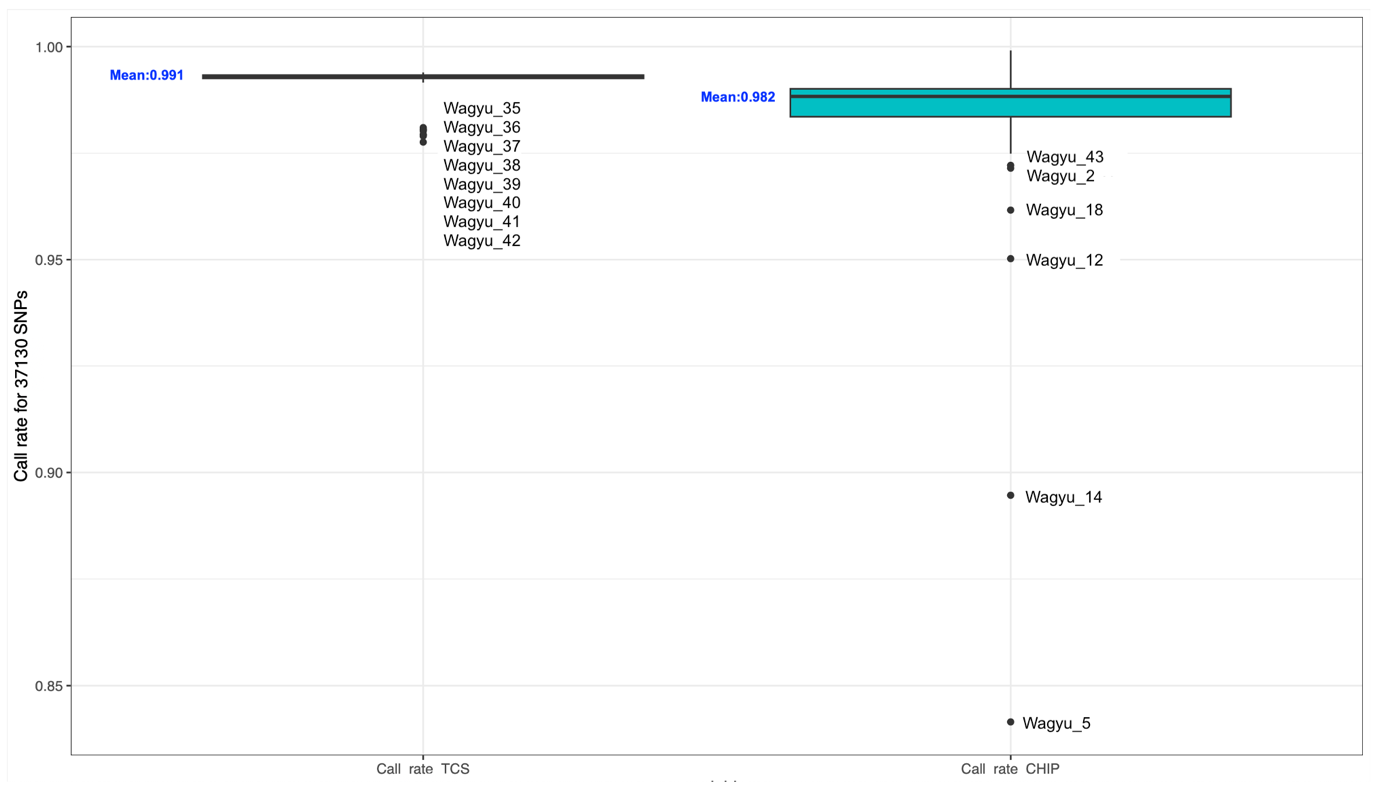


**Figure S4. Comparison of the call rates of TCS and Versa50K at the sample level using 37130 SNPs that are commonly detected.** TCS has a significantly higher (T-test: p-value = 1.309e-02) call rate than Versa50K, with a mean call rate of 0.991. Samples Wagyu_5, Wagyu_14, Wagyu_12, Wagyu_18, Wagyu_2, and Wagyu_43 are outliers with a low call rate in Versa50K, whereas the mean call rate is 0.983.
