# Supplementary material for: Evaluating the Efficacy of Target Capture Sequencing for Genotyping in Cattle
Source: Genes (Basel). 2024 Sep 18;15(9):1218. doi: 10.3390/genes15091218 (PMC11431841; doi:10.3390/genes15091218)
Supplement: Supplementary file 1 [file genes-15-01218-s001.zip › Probe_capture_paper_supplementary_files_20240910/Sub_Figures/FigureS10_seq_depth_distribution_30X.docx]

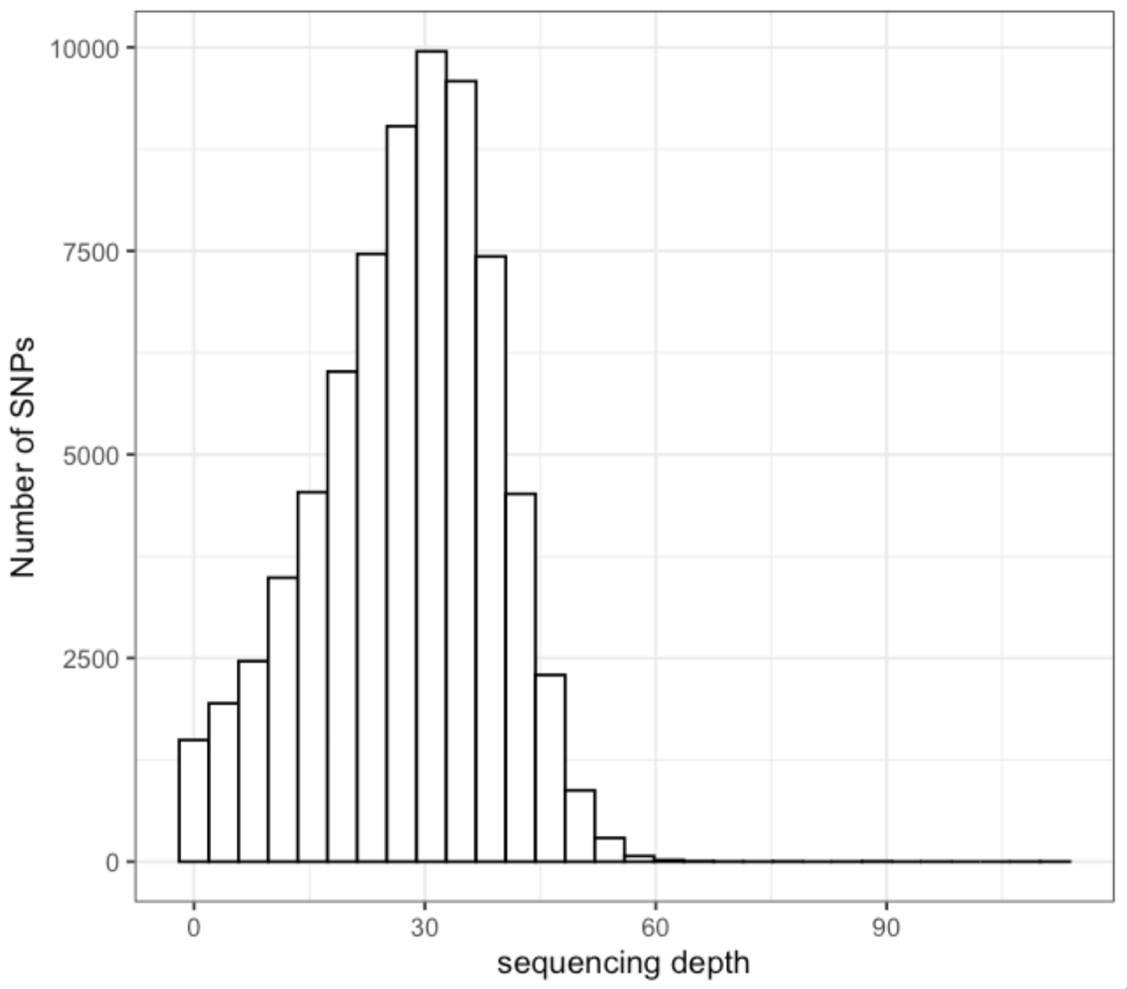


**Figure S10. The distribution of average sequencing depth for 71553 SNPs crossing 54 samples (two sample was removed as low quality for Versa50K) after down sampling for 30X.**
