# Supplementary material for: Evaluating the Efficacy of Target Capture Sequencing for Genotyping in Cattle
Source: Genes (Basel). 2024 Sep 18;15(9):1218. doi: 10.3390/genes15091218 (PMC11431841; doi:10.3390/genes15091218)
Supplement: Supplementary file 1 [file genes-15-01218-s001.zip › Probe_capture_paper_supplementary_files_20240910/Sub_Figures/FigureS3_SNPwise_concordance.docx]

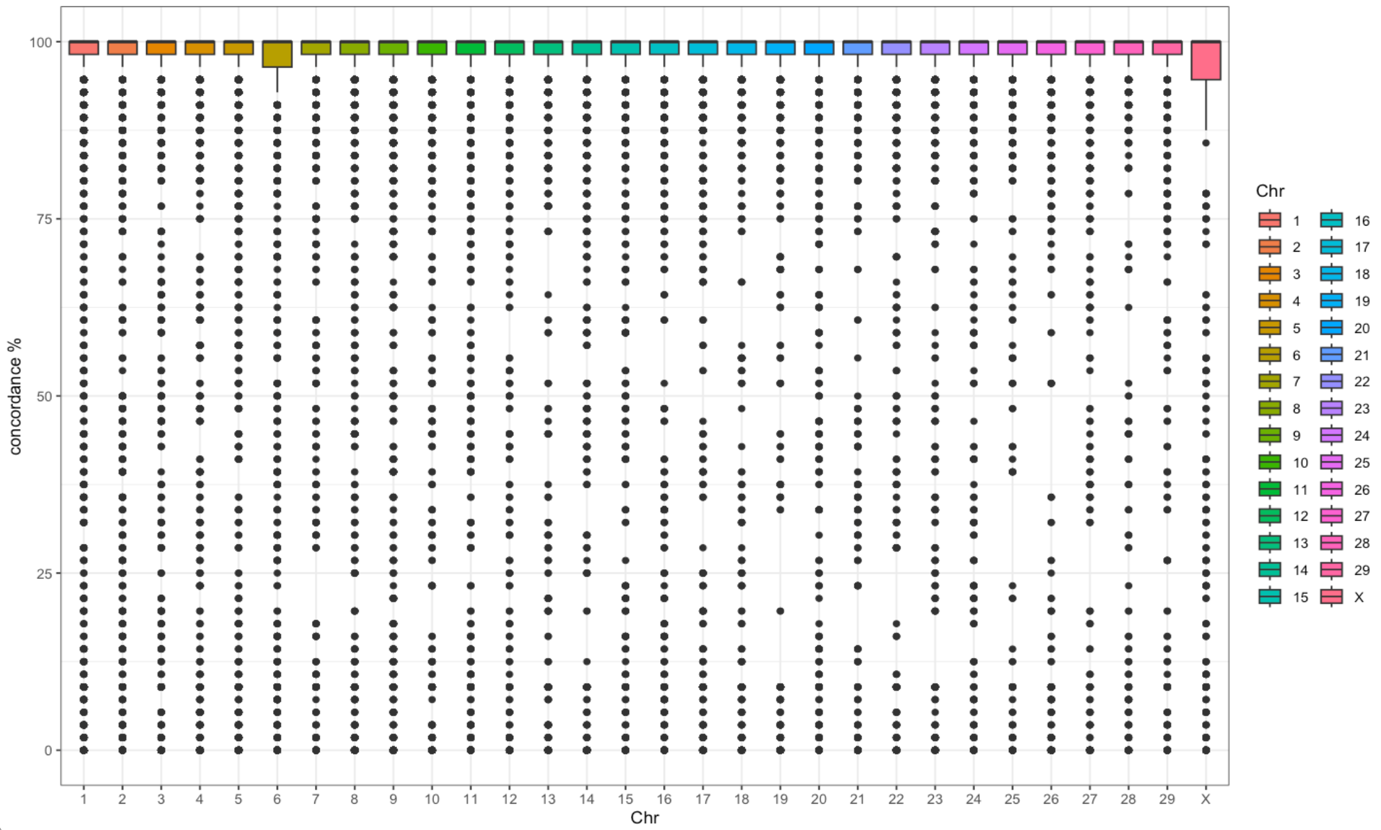


**Figure S3. The concordance of SNPs called by GATK and BCFtools on each chromosome.** The genotyping concordance between target captured sequencing and SNP array for SNPs called on different chromosomes. Y chromosome with only three SNPs are not listed. The concordance of SNPs on Y chromosome can be found in table S10.
